# Structural insights into signal transduction in photoactivated adenylyl cyclase through molecular dynamics simulations

**DOI:** 10.1002/pro.70830

**Published:** 2026-11-05

**Authors:** Akiya Fukuda, Masahiko Taguchi, Justin Chan, Shun Sakuraba, Eriko Nango, Hidetoshi Kono

**Affiliations:** ^1^ Graduate School of Science Tohoku University Sendai Miyagi Japan; ^2^ Institute of Multidisciplinary Research for Advanced Materials Tohoku University Sendai Miyagi Japan; ^3^ Institute for Quantum Life Science National Institutes for Quantum Science and Technology Chiba Japan; ^4^ Center of Quantum Life Science for Structural Therapeutics Chiba University Chiba Japan; ^5^ International Center for Synchrotron Radiation Innovation Smart Tohoku University Sendai Miyagi Japan

**Keywords:** BLUF domain, molecular dynamics simulation, multi‐domain protein, photoactivated adenylyl cyclase, signal transduction

## Abstract

OaPAC, from *Oscillatoria acuminata*, is a homodimer protein consisting of the blue‐light using flavin domain and the adenylyl cyclase (AC) domain, linked by long coiled‐coil α3‐helices. Upon blue‐light absorption, a rearrangement of the hydrogen‐bonding network involving the flavin chromophore, Y6, and Q48 triggers signal propagation through the α3‐helices to the AC domain. While the recent crystallographic structure of the W90–M92‐switched conformation in the light state provided significant structural information, the underlying atomistic mechanism of signal transduction remains unclear. In this study, we performed molecular dynamics simulations of OaPAC for four models: wild‐type (WT) dark state, W90–M92 unswitched WT light state, W90–M92 switched WT light state, and W90–M92 unswitched L111A/L115A light state. Our results show that the switched WT light‐state model exhibits structural fluctuation approximately twice as large as that in the unswitched WT light state. Furthermore, we identified a pronounced transition between two distinct conformational states in the switched WT light—“open” and “closed” —characterized by the angle between the α3‐helices and the “handle” region. Notably, the occupancy of the closed conformation was highest in the switched WT light state. In contrast, the non‐functional L111A/L115A mutant remained trapped in a rigid open conformation, reflecting its loss of activity and linking structural flexibility to enzymatic competence. These findings suggest that the W90–M92 conformational switch is a critical, multi‐step trigger of OaPAC signal transduction, inducing a transient asymmetric (open–closed) conformation and a flexion motion that may facilitate enzymatic activation. This study would provide key atomistic insights for the rational design of advanced optogenetic actuators.

## 1 INTRODUCTION

Cyclic adenosine monophosphate (cAMP) is one of the most versatile and ubiquitous second messengers, playing a fundamental role in transducing extracellular signals into diverse intracellular responses. Its capacity to regulate key processes—such as the activation of protein kinase A (Johnson et al., 2001), gene expression (Mayr & Montminy, 2001), and ion channel activity (Wainger et al., 2001)—is highly dependent on its intracellular concentration. Adenylyl cyclase (AC) was first identified as an enzyme that produces cAMP from adenosine triphosphate (ATP) (Sutherland, 1972). A distinct class of transmembrane ACs regulated by G‐proteins was subsequently characterized (Krupinski et al., 1989). In contrast, photoactivated adenylyl cyclase (PAC) enables the production of cAMP in response to light stimulation, bypassing the requirement for G‐proteins. PAC has therefore emerged as a powerful tool for the precise spatiotemporal control of cellular functions, with applications ranging from neuroscience to cardiology (Hagio et al., 2023; Sitjà‐Roqueta et al., 2025; Vittani et al., 2025).

Although PAC was identified as a light avoidance sensor protein in *Euglena* in 2002 (Iseki et al., 2002), its atomic‐level structure remained elusive for over a decade. OaPAC, from *Oscillatoria acuminata*, is a homodimeric multi‐domain protein with C2 symmetry, in which each monomer (366 amino acids) consists of a BLUF domain (residues 1–102) and an AC domain (residues 127–366) connected via coiled‐coil α3‐helices (residues 103–125) and an α4‐helix (residues 127–143) termed the “handle” (Figures 1 and S1). In 2016, Ohki et al. reported the first atomic‐resolution x‐ray crystal structure of OaPAC in the dark state (Ohki et al., 2016), followed by the light‐state structure (Ohki et al., 2017). The structure of *Beggiatoa* PAC (bPAC), which shares ~57% sequence homology with OaPAC but exhibits approximately 100‐fold higher enzymatic activity, was also determined (Lindner et al., 2017). More recently, serial femtosecond x‐ray crystallography (SFX) using an x‐ray free‐electron laser (XFEL) provided the structure of OaPAC bound to substrate ATP (Chretien et al., 2024) and a Y6W mutant (Kapetanaki et al., 2024). Despite these structural advances, the molecular mechanism by which light absorption at the BLUF domain triggers catalytic activation of the AC domain remains incompletely understood.

**FIGURE 1:**
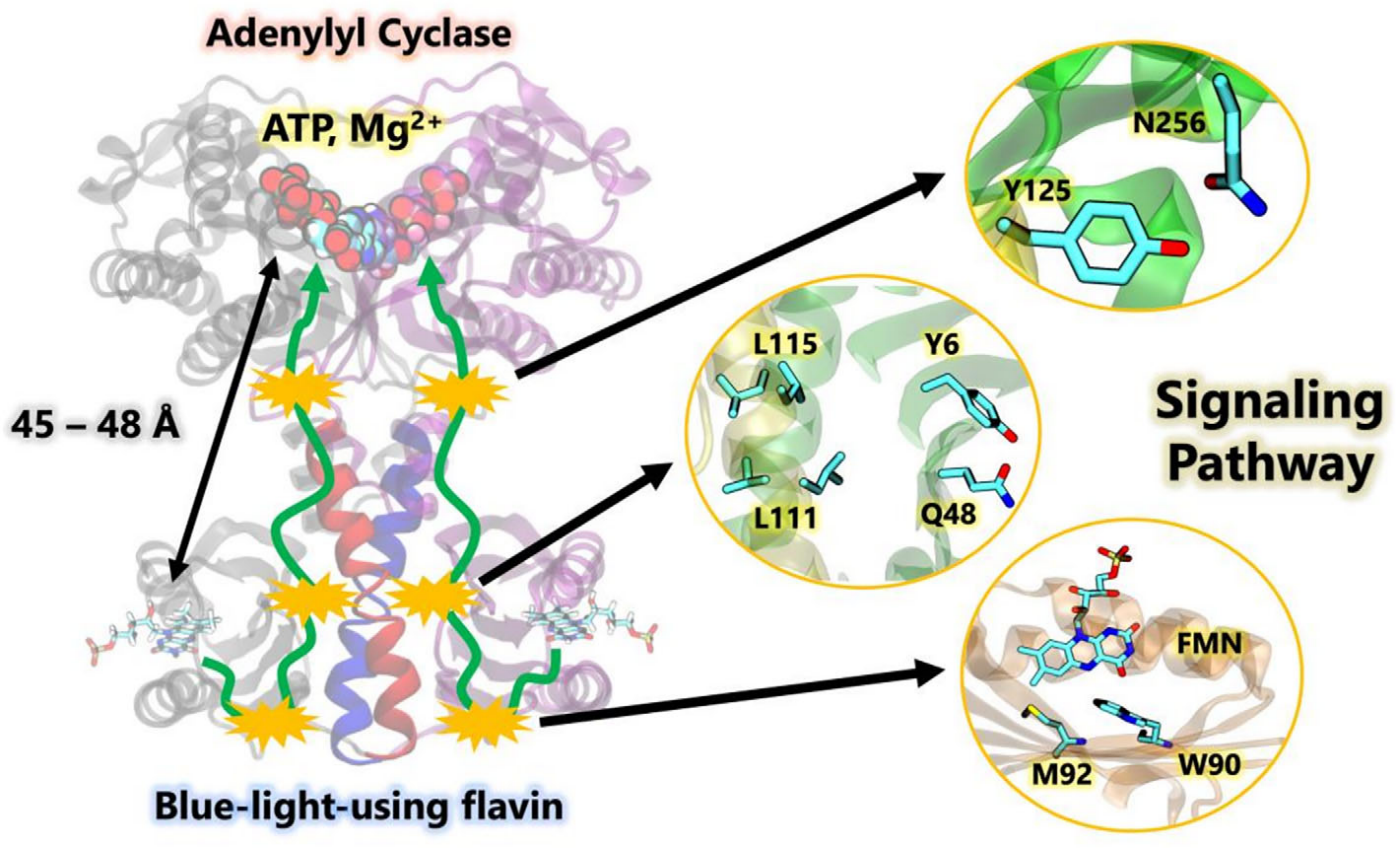
Overall structure of OaPAC and the proposed signaling pathway. The homodimeric structure of OaPAC consists of the blue‐light‐using flavin (BLUF) domain and the adenylyl cyclase (AC) domain. The distance between the chromophore (flavin mononucleotide [FMN]) and the active site (adenosine triphosphate [ATP], Mg^2+^) is approximately 45–48 Å, indicating an allosteric regulation mechanism. Green arrows indicate the proposed signaling pathway propagating from the BLUF domain to the AC domain through the central α3‐helices. The circular insets highlight key residues involved in this structural transduction: (bottom) FMN, W90, and M92, which are crucial for the photoactivation and the W90–M92 switch; (middle) L111 and L115, essential for the stable dimerization of the α3‐helices; and (top) Y125 and N256, which form a critical interaction network at the interface between the α3‐helix and the AC domain.

The enzymatic activity of PAC is regulated by the BLUF domain, which utilizes flavin adenine dinucleotide (FAD) or flavin mononucleotide (FMN) as a chromophore (Figure 1). Extensive research has focused on the photoactivation mechanism of the BLUF domain (Anderson et al., 2005; Collado et al., 2022; Grinstead et al., 2006; Hasegawa et al., 2004; Iwata et al., 2018; Jewlikar et al., 2025; Masuda et al., 2004, 2005, 2008; Mathes et al., 2011; Taguchi et al., 2024; Tokonami et al., 2022; Zhou et al., 2022). Spectroscopy and x‐ray crystallography have shown that blue‐light illumination rearranges the hydrogen‐bonding network of the flavin chromophore, Y6, and Q48 (AppA: Y21, Q63; Slr1684: Y8, Q50; BlrB: Y9, Q51), resulting in a characteristic 10–15 nm red shift in the absorption spectrum (Hasegawa et al., 2004; Kapetanaki et al., 2024; Masuda et al., 2004, 2005; Ohki et al., 2016). The role of Q48 amide‐imidic acid tautomerization and side chain rotation has been a subject of long‐standing debate (Domratcheva et al., 2008, 2016; Gauden et al., 2006; Hontani et al., 2023; Iwata et al., 2018; Lukacs et al., 2011; Sadeghian et al., 2008; Stelling et al., 2007; Unno et al., 2006); our quantum mechanics/molecular mechanics (QM/MM) free‐energy calculations demonstrated that a combination of tautomerization and side chain rotation is energetically more stable by ~4.7 kcal/mol than tautomerization alone (Taguchi et al., 2024), a finding subsequently supported by TR‐SFX capturing Q48 rotation on a microsecond timescale (Chretien et al., 2024). The roles of the semi‐conserved W90 and well‐conserved M92 residues (AppA: W104, M106; PixD: W91, and M93) have also been investigated (Bonetti et al., 2009; Collado et al., 2022; Hall et al., 2019; Masuda et al., 2008; Mehlhorn et al., 2015; Wu & Gardner, 2009; Yuan et al., 2006). Tokonami et al. identified two millisecond‐timescale phases in BLUF photoactivation: a faster phase involving local core rearrangements and a subsequent phase in which W90 plays a pivotal role in propagating the signal toward the C‐terminus (Tokonami et al., 2022). Consistent with this, Chretien et al. captured the crystal structure of the W90–M92 switched conformation by cryo‐trapping SFX (Chretien et al., 2024): in this light‐state conformation, W90 moves toward the BLUF hydrophobic core—closer to the flavin—while M92 shifts away from the isoalloxazine ring, suggesting that this structural switch initiates signal propagation through the α3‐helices to the AC domain.

In OaPAC, the BLUF photoreceptor region and the AC catalytic region are separated by approximately 45–48 Å, precluding direct interaction (Figure 1). Signal transmission, therefore, presumably occurs through the α3‐helices. In addition, Nakasone et al. (2023) showed that OaPAC requires excitation of both BLUF domains for full enzymatic activity. Ohki et al. (2016) demonstrated that the L111A/L115A mutation in the α3‐helix—essential for dimerization—completely abolishes catalytic activities in both dark and light states. Since single alanine substitutions at either L111 or L115 failed protein expression, these residues may be critical for the structural stability of the dimer interface. Also, Y125A and N256A mutations, which eliminate hydrogen bonds at the α3‐helix–AC domain interface, also reduce light‐state enzymatic activity (Ohki et al., 2016). Spectroscopy with unnatural amino acids provides additional support for their role in signal transduction (Jewlikar et al., 2025). Despite these extensive mutational and biochemical insights, the precise atomistic mechanism by which structural changes in the BLUF domain are dynamically transmitted over a distance of 45–48 Å to modulate the AC active site remains elusive.

To address this gap, we performed molecular dynamics (MD) simulations of OaPAC to provide atomic‐scale structural insights into the signal transduction mechanism. The structures and atomic charges of the photoreceptor region—specifically, the isoalloxazine ring of flavin chromophore and side chains of Y6 and Q48—were taken from our previous hybrid QM/MM free‐energy geometry optimizations (Taguchi et al., 2024). We constructed four simulation models: WT dark state, W90–M92 unswitched WT light state, W90–M92 switched WT light state, and W90–M92 unswitched L111A/L115A light state. For clarity, the conformation with the W90–M92 switch is hereafter referred to as “switched,” while the light‐state conformation without this switch is referred to as “unswitched.” Our MD simulations are thus designed to address this gap by capturing dynamic structural changes that are inaccessible to static crystallography. To the best of our knowledge, this study is the first comprehensive MD simulation of PAC.

## 2 RESULTS

### 2.1 Overall structural dynamics of OaPAC

The overall structure of OaPAC and the domain organization used for the following structural analyses are shown in Figure 2a, with the individual domains color‐coded for clarity. To evaluate the impact of structural and charge differences in the photoreceptor region (the isoalloxazine ring part of flavin chromophore and side chains of Y6 and Q48) previously obtained by hybrid QM/MM free‐energy geometry optimizations (Taguchi et al., 2024) on the overall structural dynamics of OaPAC, we computed the backbone (C, N, O, and Cα atoms) root‐mean‐square fluctuation (RMSF). In the BLUF domain (residues 1–102), the RMSF values showed the following order: switched WT light state > unswitched WT light state > WT dark state > unswitched L111A/L115A light state (Figure 2b). Notably, the RMSF in the switched WT light state was approximately twice that of the unswitched WT light state. A similar trend was observed in the AC domain; while the WT dark, unswitched light, and L111A/L115A light states exhibited comparable RMSF values, the switched WT light state showed a twofold increase in fluctuation. These results suggest that the light‐induced rotation of the Q48 side chain alone does not provide sufficient driving force for signal transduction; rather, the W90–M92 switched conformation is essential for the structural propagation. Furthermore, OaPAC is known to exhibit basal enzymatic activity even in the dark state (Ohki et al., 2016). Excluding the vicinity of the chromophore, structural differences between the WT dark and unswitched light states appeared to be negligible, consistent with previous reports (Ohki et al., 2016). This suggests that initial conformational changes in the light state are confined to local events—specifically, the rotation of the Q48 side chain. The TR‐SFX structure obtained at microseconds after blue‐light irradiation indicates that the rotation of the Q48 side chain occurs within 1.8 μs (Chretien et al., 2024); consequently, capturing large‐scale domain rearrangements may be difficult using MD simulations that only account for structural and charge effects around the chromophore without the W90–M92 switch. Additionally, the RMSF of the L111A/L115A light state was slightly lower than that of the WT dark state, consistent with enzymatic assays showing that this mutation resulted in lower activity than the WT dark state (Ohki et al., 2016).

**FIGURE 2:**
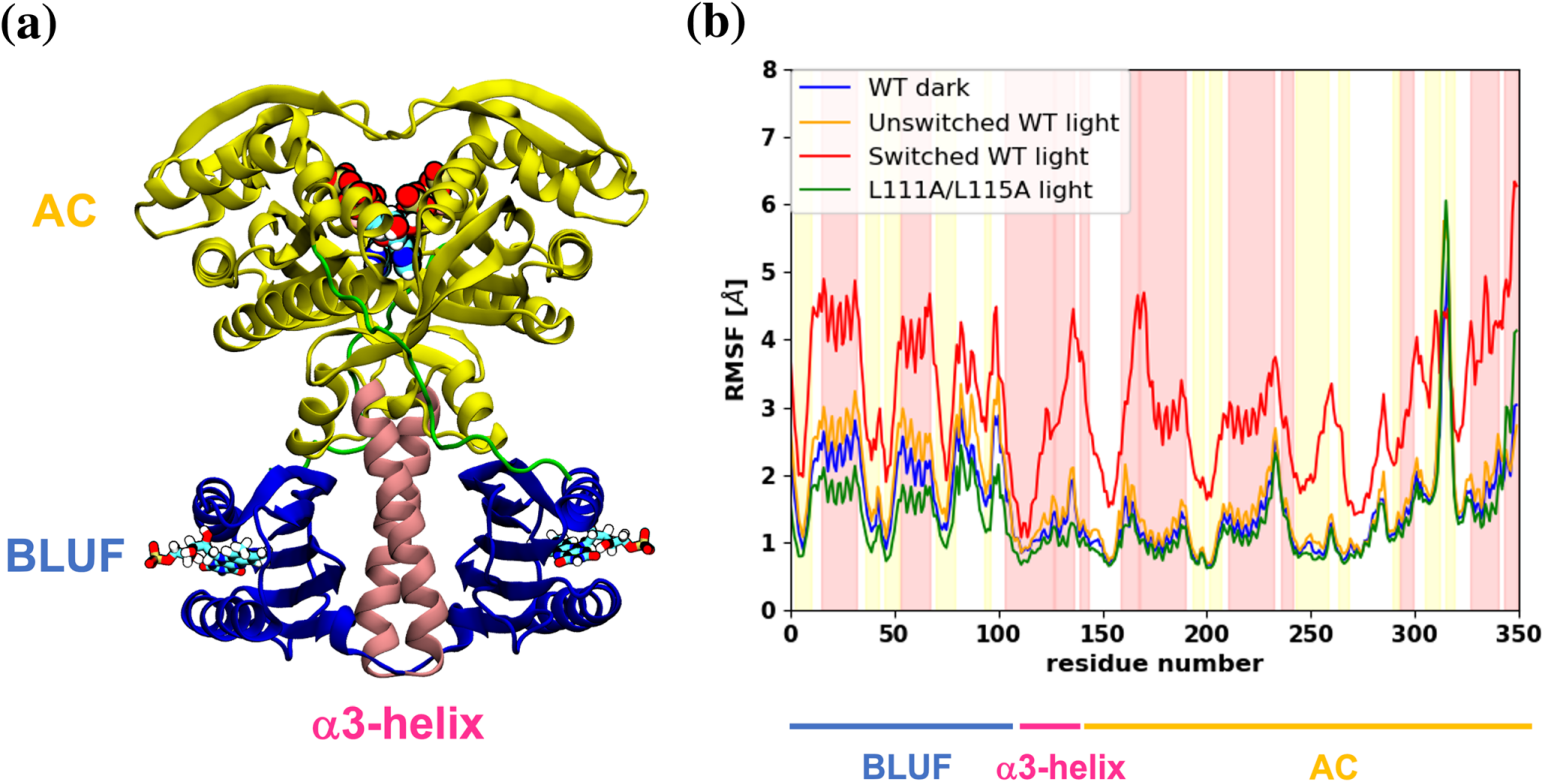
Simulation model and the result of root‐mean‐square fluctuation (RMSF). (a) Simulation model of the wild‐type (WT) dark state based on the 4YUS structure (Ohki et al., 2016). The blue‐light‐using flavin (BLUF) core, α3‐helices, adenylyl cyclase (AC), and C‐terminus are colored in *blue*, *pink*, *yellow*, and *green*, respectively. Flavin mononucleotide (FMN) is drawn as the stick. Adenosine triphosphate and Mg^2+^ are depicted in the van der Waals representation. (b) RMSF of backbone (C, N, O, and Cα) in each model is shown. The WT dark, unswitched WT light, unswitched L111A/L115A light, and switched WT light are colored in *blue*, *orange*, *green*, and *red*, respectively. The background of the RMSF plot is shaded in yellow and red to indicate sheet and helix structures, respectively.

To further characterize the nature of these structural fluctuations in the full‐length OaPAC structure, we analyzed secondary structure occupancy across the MD trajectories (Figure S2a). Previous circular dichroism studies in solution reported minimal changes in secondary structure between the dark and light states (Nakasone et al., 2023), and our simulations confirmed this, showing nearly identical secondary structure profiles across all models throughout the full‐length protein. However, in the switched WT light state, an increase in disordered regions (represented in white) was observed in the C‐terminus (residues 325–350). We next focused on the BLUF domain (residues 1–102) to identify local structural changes associated with blue‐light illumination and calculated the sum of squared differences (SSD) in secondary structure occupancy (Figure S2b). Using the WT dark state as the reference, the switched WT light state exhibited the most pronounced differences within the BLUF domain. In contrast, the unswitched WT light and L111A/L115A light states showed only marginal variations, suggesting that the unswitched light state remains structurally similar to the dark state. Residues with an SSD greater than 0.5 relative to the WT dark state were visualized to represent regions exhibiting significant changes (Figure S3). These residues were primarily located in the β4–β5 and β5–α3 linkers of the BLUF domain. Similar results were obtained when using the L111A/L115A light state as the reference (SSD >0.1), with the most variable residues occupying the same regions. Taken together, these results suggest that OaPAC activation does not involve large‐scale secondary structure transitions in the full‐length protein but rather involves localized structural changes within specific regions of the BLUF domain, consistent with solution‐state spectroscopy findings (Kapetanaki et al., 2024; Nakasone et al., 2023).

### 2.2 Conformational transition of the α3‐helices and handle region

In the switched WT light state, the angle between α3‐helices (residues 103–125) and handle region (residues 127–143) exhibited significant transitions between distinct conformations: open ($\theta >40^{{}^{\circ}}$) and closed ($\theta <40^{{}^{\circ}}$) conformations (Figure 3a). This angle, defined as illustrated in Figure 3b, was calculated across the MD trajectories using CPPTRAJ (Case et al., 2020) (Figure 3c). The angle distribution for the switched WT light state was broadly dispersed, whereas the unswitched WT light state remained centered around the values of the two crystal structures (Protein Data Bank [PDB] ID: 4YUS and 8QFG). In the WT dark state, the distribution was primarily clustered around the open conformation of the 4YUS structure, with minimal sampling of the closed conformation of the 8QFG structure. Notably, the L111A/L115A light state localized as the open conformation, with no transitions observed toward the closed conformation. The order of range for the angle distribution is consistent with that of the RMSF values reported in the previous section. Furthermore, the switched WT light state can access a wide range of angular states, indicating that the W90–M92 switch triggers significant transient dynamics (Figure 3c). While the WT dark and unswitched light states showed occasional transitions to the closed conformation, they remained predominantly in the stable open conformation. These quantitative occupancy data (Table 1) further support the conclusion that the W90–M92 switch induces the large‐scale structural rearrangements necessary for OaPAC signal transduction, underscoring its essential role.

**FIGURE 3:**
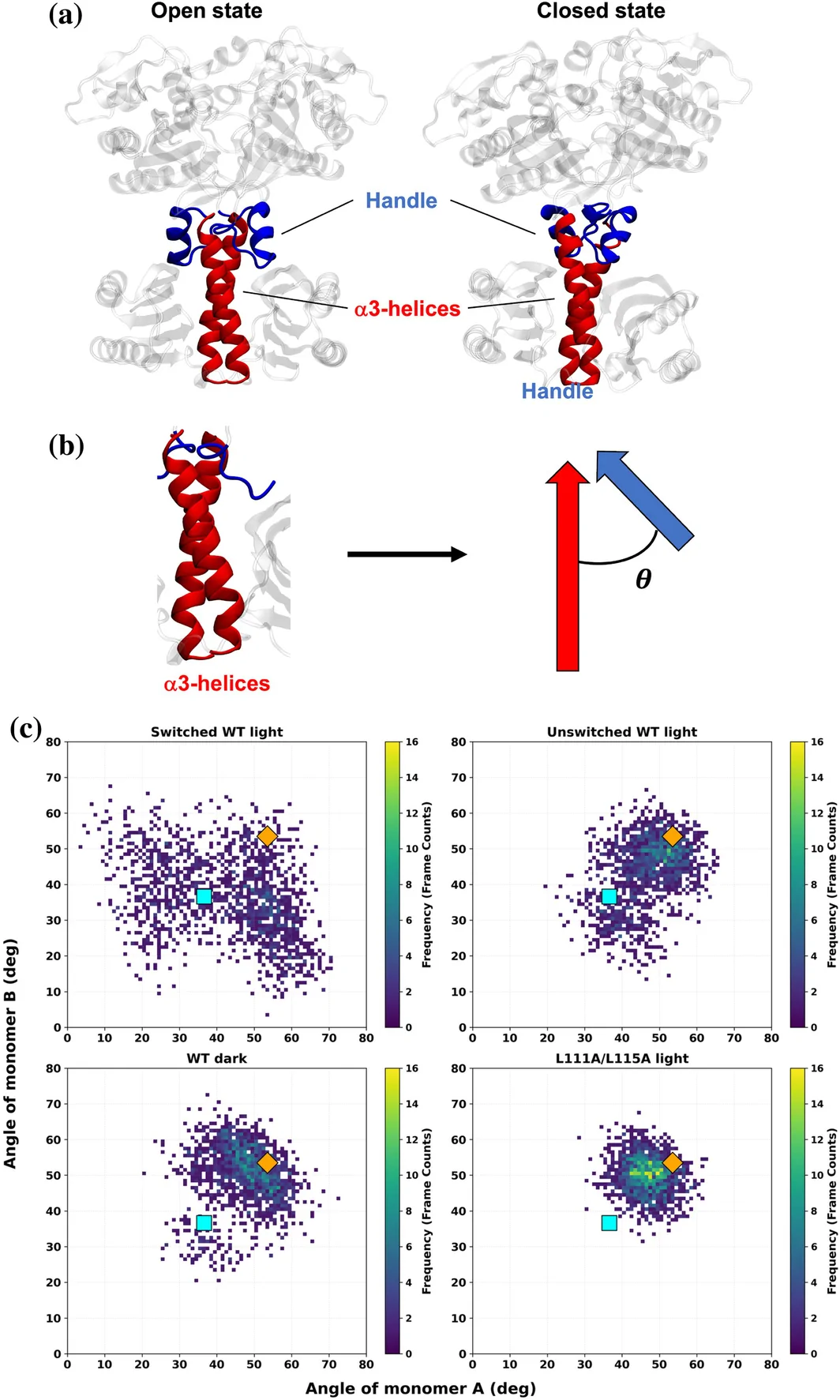
Two conformations of the angle between α3‐helices and handle, the definition of the angle for analysis, and the distribution of the angle between α3‐helices and handle in each model. (a) Snapshots of two conformations in the molecular dynamics trajectory of wild‐type (WT) light after switching. The left figure is the open conformation, and the right one is the closed conformation. α3‐helices and handle are colored in *red* and *blue*, respectively. (b) The definition of the angle between α3‐helices and the handle for analysis. The red arrow is the α3‐helices, and the blue arrow is the loop of the handle (residues 127–143). (c) The distribution of the angle between α3‐helices and the handle. Starting from the top left, you will see the switched WT light, followed by the unswitched WT light, and finally the WT dark and the L111A/L115A light. The *orange* rhombus and square represent the angles of 4YUS (53.5°; Ohki et al., 2016) and 8QFG (36.6°; Chretien et al., 2024), which are x‐ray crystallographic structures. We defined angles above 40° as the open conformation and those below 40° as the closed conformation.

**TABLE 1:** Ratio of angle states for each structural model. Values denote percentages (%).

	WT dark	Unswitched WT light	Unswitched L111A/L115A light	Switched WT light
Open–open	76.9	64.7	90.0	17.2
Open–closed	17.8	22.9	9.8	63.7
Closed–closed	5.3	12.4	0.1	19.1

Abbreviation: WT, wild‐type.

### 2.3 Hydrogen‐bond rearrangement drives β‐sheet shifting and interdomain fluctuations

#### 2.3.1 Rearrangement of hydrogen‐bonding networks and β‐sheet shifting

To investigate how the characteristic β‐sheet rearrangements seen in the switched crystal structure (PDB ID: 8QFG; Chretien et al., 2024) dynamically govern the open/closed transitions observed in Section 2.2, we analyzed the changes in hydrogen‐bonding patterns using the Baker–Hubbard identification method (Baker & Hubbard, 1984) implemented in the MDTraj Python library (McGibbon et al., 2015). Our analysis confirmed that the C‐terminal shifts of the β5‐sheet seen in the crystal structure remain stable throughout the simulations (Figure S4).

Comparison of intramonomer hydrogen bonds between the closed conformation of the switched WT light state and the open conformation of the unswitched WT light state revealed distinct changes of cluster shown in the β2‐ and β5‐sheets region (β2: residues 35–42, β5: residues 89–97) (Figure 4a, left). This pattern was not unique to the switched state; similar shifts in the backbone hydrogen‐bonding patterns were observed when comparing the unswitched WT light state with the WT dark state (Figure 4b). These altered patterns stem from a longitudinal shift of the β5‐sheet toward the C‐terminus by approximately two residues. This shift appears to drive the larger structural fluctuations observed in the RMSF analysis. These rearrangements further impact the β4‐sheet (residues 72–80), which interacts directly with the handle region. In the switched WT light state, the β4‐sheet also shifts toward the C‐terminus relative to other models. Therefore, we propose that the W90–M92 switch, combined with hydrogen bond remodeling between the β2‐ and β5‐sheets, drives the β5‐sheet shift, thereby subsequently facilitating the conformational transition of the handle region.

**FIGURE 4:**
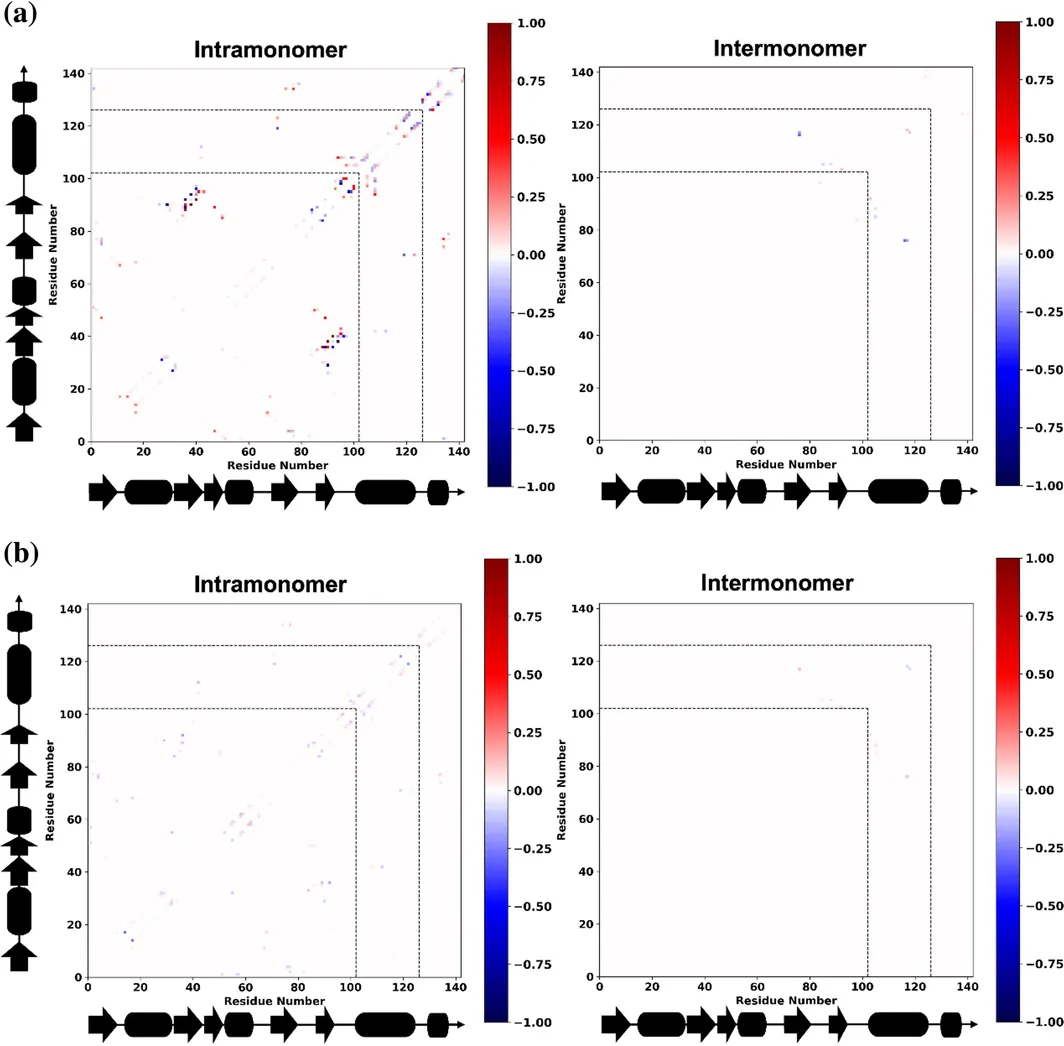
Heatmap of the difference in hydrogen‐bond analysis. Along each axis, arrows and cylinders denote β‐sheets and α‐helices, respectively. Dashed lines demarcate the blue‐light‐using flavin core (residues 1–102), the α3‐helix (residues 103–125), and the handle (residues 127–143). (a) Difference between the closed conformation in the switched wild‐type (WT) light state and the open conformation in the unswitched WT light state; the left and right panels show the intramonomer and intermonomer differences, respectively. Red indicates hydrogen bonds in the closed (switched WT light) conformation, and blue indicates those in the open (unswitched WT light) conformation. (b) Difference between the WT dark and unswitched WT light states, shown in the same layout as (a).

#### 2.3.2 Intermonomer interactions and structural fluctuations

Analysis of intermonomer hydrogen bonds revealed an increased frequency (+0.25) between the backbone nitrogen of K77(A) and the side chain oxygen of S118(B) in the unswitched WT light state compared to the WT dark state (Figure S5). In contrast, comparing the closed conformation (switched WT light) to the open conformation (unswitched WT light) showed a significant decrease in hydrogen‐bond frequency (−0.29) between K77(A) and E117(B), residues whose side chains typically form a salt bridge (Figure S6a). To understand why these changes were specific to the switched state, we analyzed the distance between the K77‐NZ and E117‐OE1/OE2 atoms (Figure S6b). The distance distributions for the WT dark and unswitched WT light states were almost similar, both favoring hydrogen bond formation. However, in the switched WT light state, this interaction is significantly disrupted, precluding hydrogen bond formation. Interestingly, in the unswitched L111A/L115A light state, the distribution remained concentrated at the hydrogen‐bonding distance, in contrast to that in the switched WT light structure.

These results suggest that the K77(A)–E117(B) hydrogen bond acts as a structural tether; its disruption in the switched state presumably triggers increased structural fluctuations between the BLUF core and the α3‐helices, facilitating signal transduction.

### 2.4 Contact map analysis of intramonomer and intermonomer interactions

To identify residues involved in residue–residue contacts, we computed contact maps using MDTraj (McGibbon et al., 2015) with a 4.5 Å cutoff for all atoms of the two residues.

#### 2.4.1 Comparison of the closed conformation in the switched WT light state and the open conformation in the unswitched WT light state

The contact differences were clearly concentrated (Figure 5a). In the intramonomer analysis, significant changes were observed in the interactions between the β4‐sheet and the α4‐helix, presumably reflecting the conformational transition of the handle. Additionally, we identified interaction changes in the α3‐helices—both intramonomer and intermonomer—that were not detectable in the hydrogen‐bond analysis. Specifically, contacts between the α3‐helix and the β2/β3‐sheets were highly localized. These findings confirm that OaPAC signal transduction is not driven by direct interactions from the FMN molecule at each monomer. Similar to the hydrogen‐bond analysis, interactions between the β5‐sheet and the β2/β3‐sheets were also observed. Notably, due to the significant structural divergence between the initial switched and unswitched models, we focused on identifying key residues for the transitions described below.

**FIGURE 5:**
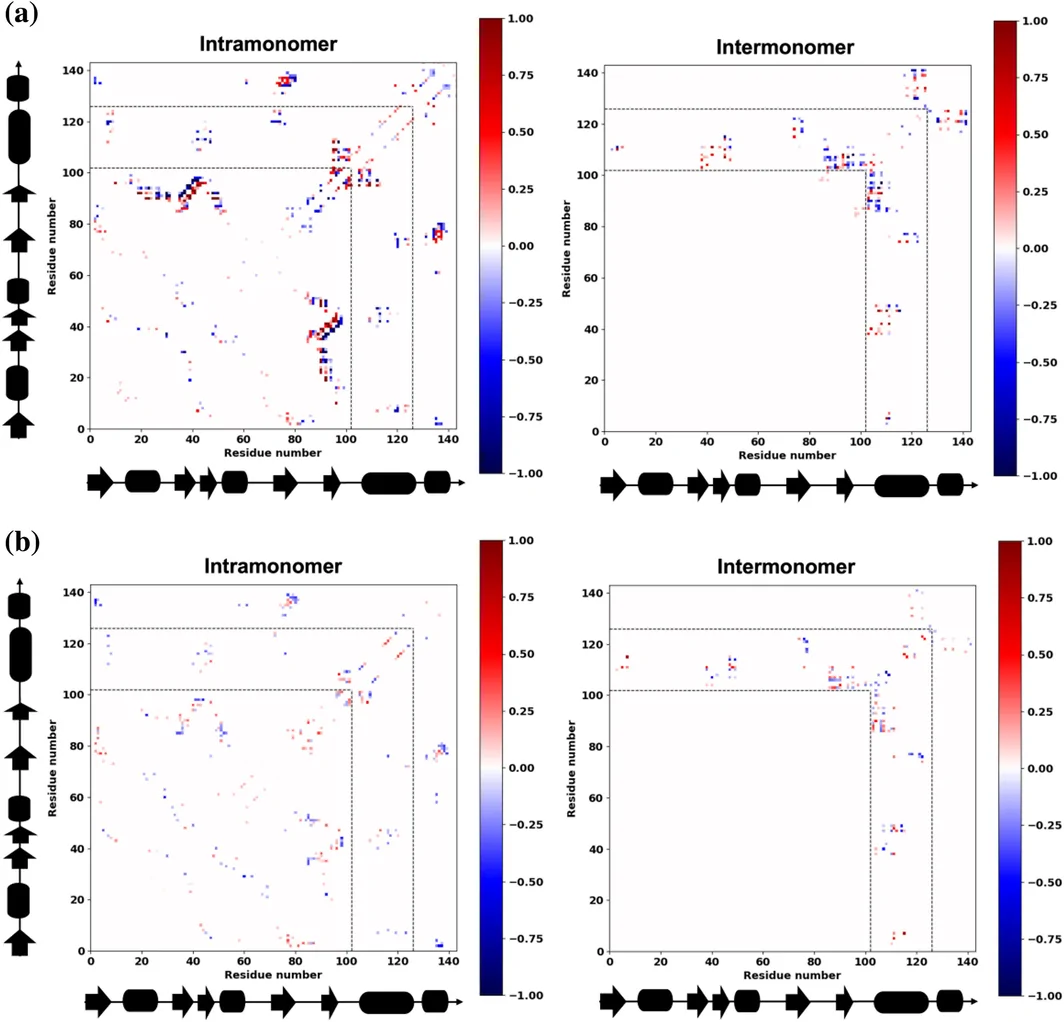
Difference contact map. Along each axis, arrows and cylinders denote β‐sheets and α‐helices, respectively; the left and right panels show the intramonomer and intermonomer differences, respectively. (a) Differences between the closed conformation in the W90–M92 switched wild‐type (WT) light state and the open conformation in the unswitched WT light state. Red indicates contacts in the closed conformation, and blue indicates those in the open conformation. (b) Differences between the W90–M92 unswitched WT light state and the WT dark state, shown in the same layout as (a).

#### 2.4.2 Structural precursors to the W90–M92 switch

To identify the structural “signatures” leading to the W90–M92 switch, we examined the difference in contact frequency between the unswitched WT light and WT dark states (Figure 5b). While in the previous section, specific trajectories were used (closed conformation vs. open conformation), in this analysis, we utilized all trajectories to capture the mixture of conformations. The trends remained consistent: changes were observed in the handle–β4, α3–β2/β3, and β5–β2/β3 interfaces. By ranking contact frequencies (Figure 6), we identified specific residue pairs that interact between the root of the α3‐helix and the β5‐sheet, which serve as precursors to the W90–M92 switch. As discussed in the hydrogen‐bond analysis section, the rearrangement of interaction patterns between the β2‐ and β5‐sheets presumably facilitates the subsequent switch. Furthermore, residues in the middle region of the α3‐helix altered their contacts with the β1‐, β2‐, and β3‐sheets.

**FIGURE 6:**
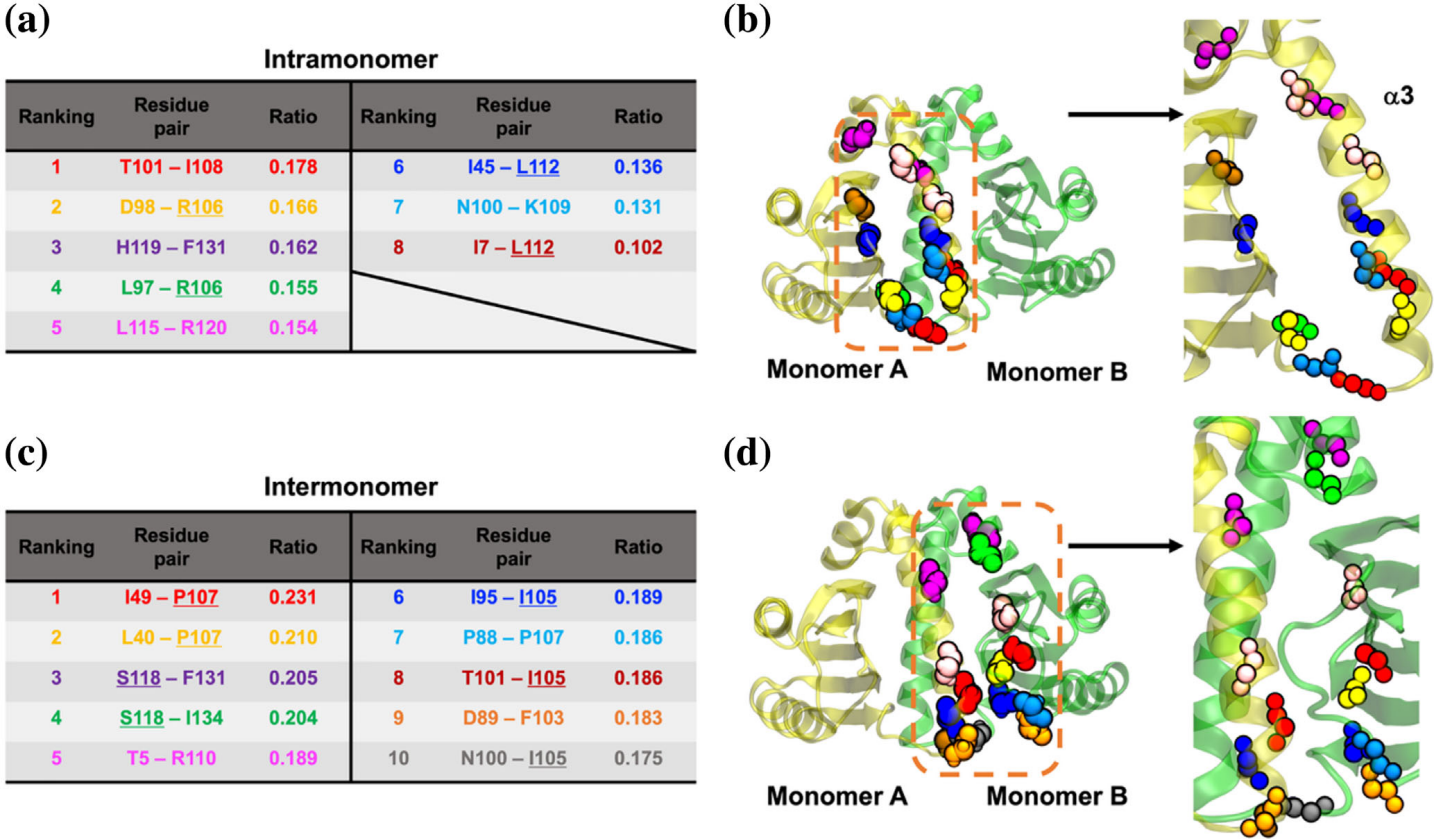
Analysis of residue‐wise contacts focused on the α3‐helix (residues 103–125) in the intramonomer and intermonomer of the unswitched wild‐type (WT) light state. (a) Ranking of the difference in contact frequencies between the WT dark and unswitched WT light states in the intramonomer. Underlining indicates residue pairs observed at least twice. (b) The unswitched WT light model averaged over the 15 μs trajectory; the protein backbone is shown as a ribbon, and the backbone atoms of the residues listed in a are shown in the van der Waals representation. (c) Ranking of the difference in contact frequencies between the WT dark and unswitched WT light states in the intermonomer. Underlining indicates residue pairs observed at least twice. (d) The backbone atoms of the residues listed in (c), shown in the van der Waals representation.

Concurrently, K77 (on the β4‐sheet) may modulate intramonomer interactions with the handle region (Figure 7). As described in Section 2.3, the K77‐E117 salt bridge is abruptly disrupted upon the W90–M92 switch; in the contact map, this local event amplifies the interaction between the β4‐sheet and the handle region.

**FIGURE 7:**
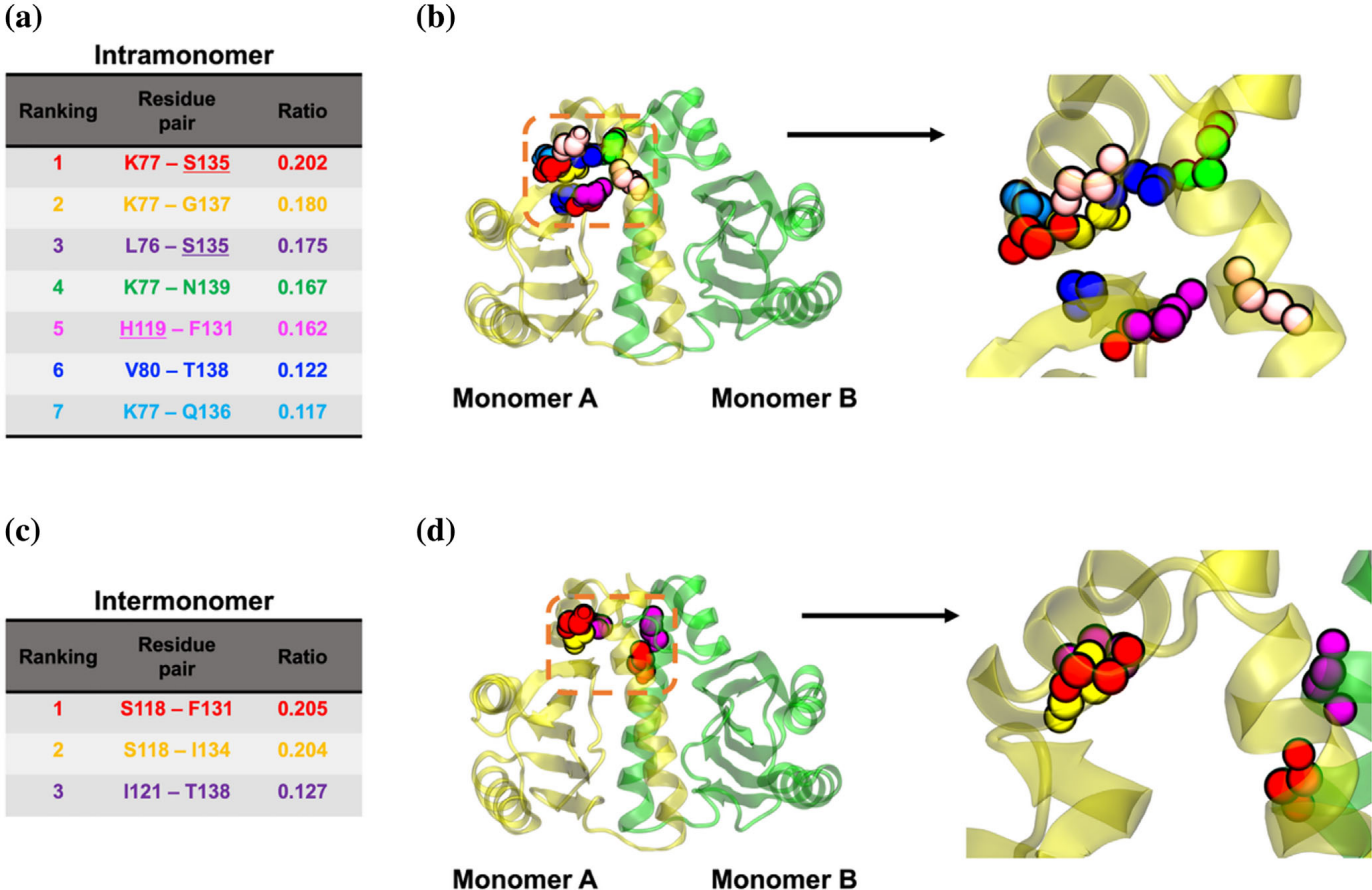
Analysis of residue‐wise contacts focused on the handle (residues 127–143) in the intramonomer and intermonomer of the unswitched wild‐type (WT) light state. (a) Ranking of the difference in contact frequencies between the WT dark and unswitched WT light states in the intramonomer. Underlining indicates residue pairs observed at least twice. (b) The unswitched WT light model averaged over the 15 μs trajectory; the protein backbone is shown as a ribbon, and the backbone atoms of the residues listed in a are shown in the van der Waals representation. (c) Ranking of the difference in contact frequencies between the WT dark and unswitched WT light states in the intermonomer. Underlining indicates residue pairs observed at least twice. (d) Backbone atoms of the residues listed in (c), shown in the van der Waals representation.

### 2.5 Residue‐level dynamics underlying signal transduction

#### 2.5.1 Role of the β5–α3 linker and M92 dynamics

The linker (residues 97–102) between the β5‐sheet and the α3‐helix underwent significant changes in the switched light state structure (Figure S7). Specifically, L97 showed increased intramonomer contacts with N100 and R106, while its interactions with F47, T101, and I105 decreased (Figure S7a,b). Secondary structure analysis confirmed that the helical and bend fractions of this linker increase in the switched state, whereas they decrease in the L111A/L115A mutant (Figure S7c). M92 dynamics also exhibited clear differences between the dark and unswitched light states (Figure S8a). In the light state, the M92 side chain increased its contact with I22, while decreasing its contacts with V35, T36, and I49. Quantitatively, the peak of the distance distribution between the M92‐CE atom and the I22‐O atom shifted from 8 Å in the WT dark state to 5 Å in both the unswitched WT light and L111A/L115A light states (Figure S8c), which correlates with the altered dihedral angle distribution of M92 (Figure S8d). Furthermore, distance analysis between W90 and P107 (at the root of the α3‐helix) revealed that while the overall distributions for the WT dark and unswitched WT light states were largely overlapping, a specific population in the 6–7 Å range notably disappeared in the unswitched WT light state. In the L111A/L115A light state, this W90–P107 distance distribution showed a more pronounced shift toward shorter distances (Figure S9).

#### 2.5.2 Impact of the L111A/L115A mutant on stability

Finally, we characterized the L111A/L115A mutant, which exhibited the lowest overall RMSF among all simulated models (Figure 2b). Contact maps revealed that L111 and L115 are critical for internal packing: L111 forms extensive interactions with the opposing β‐sheet of the BLUF core, while L115 coordinates with I7 (on the β1‐sheet), F47 (adjacent to the Q48 switch), T116, and H119 (Figures S10 and S11). Despite the complete loss of hydrophobic bulk due to the alanine mutations, structural fluctuations were significantly suppressed, indicating induced structural rigidity in this non‐functional state.

### 2.6 Dominant dynamics and functional conformational modes identified by PCA

To understand the global nature of these residue‐level changes, we performed principal component analysis (PCA) on the backbone atoms (C, N, O, and Cα atoms). To compare dynamics across models within a consistent framework, the eigenvalues of each model were scaled by the cosine similarity between its corresponding eigenvectors and those of the switched WT light state (see Section 4).

In this analysis, we focused on the top three modes: $\lambda_{1}$ corresponds to a flexion movement (Figure S12, Movie S1), where the inter‐protomer distance between the BLUF and AC domains gradually decreases; $\lambda_{2}$ represents a twisting movement of the BLUF‐AC domains (Figure S12, Movie S2); and $\lambda_{3}$ involves an opening motion of the AC domains (Figure S12, Movie S3). Interestingly, while the populations of $\lambda_{1}$ and $\lambda_{2}$ are comparable in the WT dark and unswitched light states, $\lambda_{1}$ becomes dominant in the switched WT light state, increasing by approximately 10% (Table 2). This flexion motion has been proposed as a critical factor driving conformational changes in the AC domain (Lindner et al., 2017). Consistent with the RMSF (Figure 2b), the AC domain in the switched WT light state exhibits substantial flexibility. Therefore, the dominance of the flexion mode suggests that it is a primary driver of functional conformational changes in the AC domain. In contrast, the L111A/L115A variant showed the smallest structural changes among all models (Table 2). Consequently, although the relative population of the $\lambda_{1}$ mode appears high, its absolute scaled eigenvalue remains extremely small (Table 2). This indicates that the mutation fails to induce the characteristic functional motions required to reach the transient state, thereby inhibiting enzymatic activity.

**TABLE 2:** Contributions and eigenvalues of the principal modes from principal component analysis.

	(1) WT dark	(2) Unswitched WT light	(3) Unswitched L111A/L115A light	(4) Switched WT light
Contribution (%)				
*λ*_1_	40.4	44.3	42.2	54.7
*λ*_2_	41.4	46.8	27.3	32.4
*λ*_3_	18.1	8.7	30.4	12.8
Scaled eigenvalue				
*λ*_1_	1214	3031	1143	11,632
*λ*_2_	1272	3197	740	6883
*λ*_3_	682	600	824	2713

*Note*: Columns: (1) WT dark, (2) unswitched WT light, (3) unswitched L111A/L115A light, and (4) switched WT light. The eigenvalues of Models (1–3) are scaled relative to the switched WT light state (4) using Equations (2) and (3). Mode 1 is the flexion motion (Movie S1), Mode 2 is the twisting motion between the blue‐light‐using flavin and adenylyl cyclase (AC) domains (Movie S2), and Mode 3 is the opening motion of the AC domain (Movie S3).

Abbreviation: WT, wild‐type.

## 3 DISCUSSION

The significance of the W90–M92 switch in the BLUF domain remains a controversial topic in previous studies (Bonetti et al., 2009; Collado et al., 2022; Hall et al., 2019; Masuda et al., 2008; Mehlhorn et al., 2015; Wu & Gardner, 2009; Yuan et al., 2006). The cryo‐cooled x‐ray crystallography revealed structural differences between the dark and light states: the side chain of Q48 rotates by approximately 40°, M92 moves away from Q48, and W90 undergoes only a minor conformational change (PDB ID: 5X4T; Ohki et al., 2017). However, these structural changes between the dark and light states were limited to the local environment around the flavin chromophore. Similarly, no significant global differences were observed in bPAC before and after blue‐light irradiation, which shares high homology with OaPAC (Lindner et al., 2017). In contrast, Chretien et al. successfully captured a crystallographic structure that exhibited much more pronounced structural changes following the W90–M92 switch (PDB ID: 8QFG; Chretien et al., 2024). They proposed a mechanism in which W90 enters the internal BLUF core, triggering the β5‐sheet to adopt a new conformation and shift toward the C‐terminus by approximately two residues. Compared to the dark state, the W90–M92 switched light state is markedly different, with a Cα‐root‐mean‐square deviation (RMSD) of 1.36 Å and an all‐atom RMSD of 1.93 Å. Despite these significant structural observations, the precise link between the W90–M92 switch and the downstream signal transduction mechanism remained elusive.

Our computational study strongly supports the hypothesis that the W90–M92 switch is a pivotal event driving the OaPAC signal transduction. RMSF analysis indicated that this switch induces the most significant structural fluctuations, suggesting that the mere rearrangement of the hydrogen‐bonding network around the flavin is insufficient to drive large‐scale fluctuations. Furthermore, the L111A/L115A mutation, which suppresses these fluctuations in the BLUF domain, underscores a strong correlation between structural flexibility and enzymatic activity. However, increased structural flexibility itself should not be regarded as direct evidence of catalytic competence. Rather, our results suggest that an appropriate degree of flexibility may facilitate access to specific collective motions and conformational transitions associated with signal transduction. In particular, the switched WT light state exhibited not only the largest structural fluctuations but also an increased relative contribution of the flexion mode and pronounced transitions between the open and closed conformations. Thus, the functional relevance of structural flexibility may lie in its ability to permit directional collective motions and conformational transitions associated with activation, rather than in the magnitude of the fluctuations alone. Nevertheless, the direct causal relationship between these dynamic properties and catalytic activity remains to be experimentally established. Secondary structure analysis implied that OaPAC undergoes conformational changes while maintaining overall structural order. This is consistent with solution‐state circular dichroism experiments (Nakasone et al., 2023).

In previous studies, signal transduction was thought to occur through mutual interactions among the α3‐helices, which facilitate dimerization (Nakasone et al., 2023; Ohki et al., 2016). Our study, however, reveals that OaPAC adopts two distinct conformations determined by the angle between the α3‐helices (residues: 103–125) and the handle region (residues: 127–143) (Figure 3a). Although Chretien et al. reported that Q127–S129 and F131 on the handle region shifts by approximately one residue into the tongue region of the adjacent AC monomer, they did not address the angular changes between α3‐helices and the handle region (Chretien et al., 2024). The WT dark state crystal structure (PDB ID: 4YUS; Ohki et al., 2016) corresponds to the open conformation, whereas the W90‐M92 switched light state structure (PDB ID: 8QFG; Chretien et al., 2024) represents the closed conformation. Our MD simulations demonstrated that the WT dark state can transition to the closed conformation (Figure 3), a finding consistent with the enzymatic assay, which shows that OaPAC retains a small portion of enzymatic activity even in the dark state (Ohki et al., 2016). Furthermore, in the W90‐M92 switched WT light state, the population of the asymmetric open–closed conformation was the highest among the four models, suggesting that this is a transient intermediate state (Table 1). Interestingly, hydrogen‐bonding analysis revealed no specific electrostatic interactions between the α3‐helices and the handle region, implying that their association is driven by van der Waals interactions (Figures 4 and 5). Our analysis confirmed that the hydrogen‐bonding network between the β2‐ and β5‐sheets, which shifts by about two residues due to the W90‐M92 switch (Chretien et al., 2024), remains stable throughout the MD simulations. In contrast to the crystallographic study of bPAC by Lindner et al. (2017), which highlighted a conserved hydrogen‐bond/van der Waals network coupling the β4‐sheet kink to the C‐terminal region of the α3‐helix, our simulations do not rank these specific β4–α3 contacts among the dominant interactions (Figure 6); instead, the principal α3 contacts involve its N‐terminal root and the β5‐sheet. Rather than a stable β4–C‐terminal‐α3 coupling, we find that the β4–α3 contact is dominated by the intermonomer K77–E117 tether, whose disruption—not persistence—accompanies signal propagation (Section 2.3).

Based on our MD simulations, the intermonomer interaction between K77(A) and E117/S118(B) may contribute to modulating structural fluctuations between the BLUF core and the α3‐helix, as described in the hydrogen‐bonding analysis (Section 2.3). We propose that the K77(A)–E117(B) salt bridge may act as a physical “tether” that helps maintain the rigid docking of the α3‐helix against the BLUF core. Interestingly, the abrupt loss of this tether upon the W90–M92 switch (Figure S6) suggests that disruption of this intermonomer interaction may facilitate the subsequent rearrangement of the β5‐sheet. From a mechanistic standpoint, the transition from the K77(A)–E117(B) salt‐bridge interaction to the K77(A)–S118(B) backbone‐mediated hydrogen bond in the unswitched light state may represent an early structural precursor of this destabilization, increasing the local flexibility required to prime the system for the subsequent structural “switch”. This tethering hypothesis is further supported by the behavior of the non‐functional unswitched L111A/L115A light‐state mutant. As evidenced by the suppressed fluctuations (Figures 2 and S6b), this variant remains predominantly in a rigid state in which the K77–E117 salt bridge is stabilized. This suggests that the L111A/L115A mutations disrupt the cooperative allosteric network required for light‐induced activation. Taken together, our MD simulations support a mechanistic model in which disruption of the K77–E117 salt bridge may promote localized fluctuations between the BLUF core and the α3‐helices, thereby facilitating subsequent large‐scale conformational changes toward the AC domain. However, this proposed tethering mechanism is derived from computational observations and remains to be experimentally validated.

The light‐induced structural rearrangements are primarily localized at the interfaces between secondary structural elements (Figure 5) as described in the contact map analysis (Section 2.4). Consistent with the hydrogen‐bonding analysis, the difference between the closed conformation (the W90–M92 switched WT light state) and the open conformation (the unswitched WT light state) showed significant interaction changes between the β2‐ and β5‐sheets, driven by the two‐residue shifts of the β5‐sheet (Figure 5a) (Chretien et al., 2024). Furthermore, the altered intramonomer interactions, including L97 in the β5‐α3 linker loop (residues 97–102), appear highly significant. Our finding that the switched WT light state exhibits a higher tendency for helix formation within this linker—which matches the order of structural fluctuations among the four models (Figures S7c and 2b)—suggests that the transient formation of a helical structure in this linker may serve as a mechanical trigger that accommodates the downstream two‐residue C‐terminus shift of the β5‐sheet. Concurrently, the precursor behavior observed in the M92 side chain highlights the subtle allosteric priming initiated at the flavin pocket (Masuda et al., 2008). The light‐induced rotation of M92 toward I22 is likely driven by the rearrangement of the hydrogen‐bonding network around the FMN chromophore, where the strengthening of the Q48‐flavin interaction weakens the neighboring Q48‐M92 interaction, as previously indicated (Taguchi et al., 2024). Therefore, subtle conformational transitions in the M92 side chain may serve as a trigger for the W90–M92 switch. Because M92 exhibited clear signs of this transition, we hypothesized that W90 might also show precursor behavior. Indeed, the light‐induced modulation of W90 dynamics and its severe restriction in the non‐functional mutant, L111A/L115A light structure, suggest that the local structural fluctuations of W90 are highly sensitive to the global activation state. Consequently, the W90–M92 switch presumably requires M92 to disrupt the hydrophobic core of the BLUF domain over a sustained duration; this sustained destabilization eventually crosses the energetic barrier required to trigger the large‐scale rearrangement of the β5‐sheet.

The functional importance of this hydrophobic core is further underscored by the behavior of the L111A/L115A variant. Generally, weakening such extensive hydrophobic packing by mutating a large leucine to alanine would be expected to amplify protein flexibility. Counterintuitively, however, our simulations indicated that this mutant is trapped in an abnormally rigid, non‐functional state due to collapsed internal packing. This rigidity may effectively penalize and prevent the structural fluctuations required to trigger the W90–M92 switch. This computational finding beautifully aligns with experimental reports that single L111A or L115A variants fail to express stably in cells (Ohki et al., 2016), highlighting the essential role of these residues in maintaining structural integrity and proper dimerization. Thus, we conclude that the hydrophobic core formed by L111 and L115 acts as a sophisticated mechanical conduit, where precise hydrophobic packing is strictly required to permit—rather than simply restrict—the dynamic allosteric transitions essential for OaPAC enzymatic activation.

PCA revealed that Mode 1, representing a flexion movement in which the distance between the BLUF and AC domains decreases, is the dominant structural mode in the W90–M92‐switched WT light state (Table 2; Modes 1, 2, and 3 are visualized in Figure S12, Movies [Link], [Link], respectively). Cryo‐trapping x‐ray crystallography has previously shown that the BLUF domain moves slightly away from its opposing protomer while shifting closer to the AC domain (Chretien et al., 2024). Specifically, the distance between Cα of I22 on the α1‐helix (BLUF domain) and Cα of E224 on the α7‐helix (AC domain) decreased from 40.7 to 38.7 Å compared to the dark state (PDB ID: 4YUS; Ohki et al., 2016, PDB ID: 8QFG; Chretien et al., 2024). In photoactivated bPAC, it has been suggested that this flexion movement shifts the “tongue” region toward the BLUF domain side, thereby altering the orientation of the AC domain (Lindner et al., 2017). Such a change in the relative orientation of the AC domains may facilitate rearrangement of the catalytic region into an activation‐competent conformation. Interestingly, while Mode 1 (flexion) and Mode 2 (twist) (Figure S12, Movie S1 and S2) could not be clearly distinguished in the WT dark and W90‐M92 unswitched light states, Mode 1 became the primary contributor to the structural dynamics in the switched WT light state. In contrast, the L111A/L115A light state exhibited a relatively larger contribution of Mode 3 than the WT states (Table 2). However, because the absolute scaled eigenvalues of the mutant were substantially smaller than those of the WT states, the increased relative contribution of $\lambda_{3}$ does not indicate enhanced Mode 3 motion but rather reflects its larger relative contribution within the strongly suppressed overall structural fluctuations. During the initial stages of signal transduction (~μs), flexion and twist movements appear to coexist; by contrast, the switched WT light state—modeling the post‐switch stage reached on the millisecond timescale (Tokonami et al., 2022)—is dominated by flexion. This suggests that the flexion movement is a crucial driver of the signaling process.

Based on our findings, we propose a refined signal transduction mechanism for OaPAC (Figure 8). First, blue‐light irradiation induces amide‐imidic acid tautomerization and the rotation of the Q48 side chain, which subsequently rearranges the hydrogen‐bonding network involving the flavin chromophore, Y6, and Q48 (Chretien et al., 2024; Iwata et al., 2018; Ohki et al., 2016; Taguchi et al., 2024). This rearrangement increases the conformational degree of freedom of the M92 side chain (Figure S8), enabling it to disrupt the hydrophobic core of the BLUF domain. This disruption increases structural fluctuations (Figure 2b), thereby allowing W90 to undergo a conformational change. These dynamic shifts enable the β4–β5 loop to facilitate the transition of the β5‐sheet to a new conformation via the W90–M92 switch (Chretien et al., 2024). Finally, the transition from an open to a closed conformation—characterized by the angle between the α3‐helices and the handle region—occurs alongside the flexion movement that brings the BLUF domain of one protomer and the AC domain of the other into closer proximity. These combined rearrangements ultimately drive the conformational changes in the AC domain required for the catalytic reaction (Chretien et al., 2024; Lindner et al., 2017).

**FIGURE 8:**
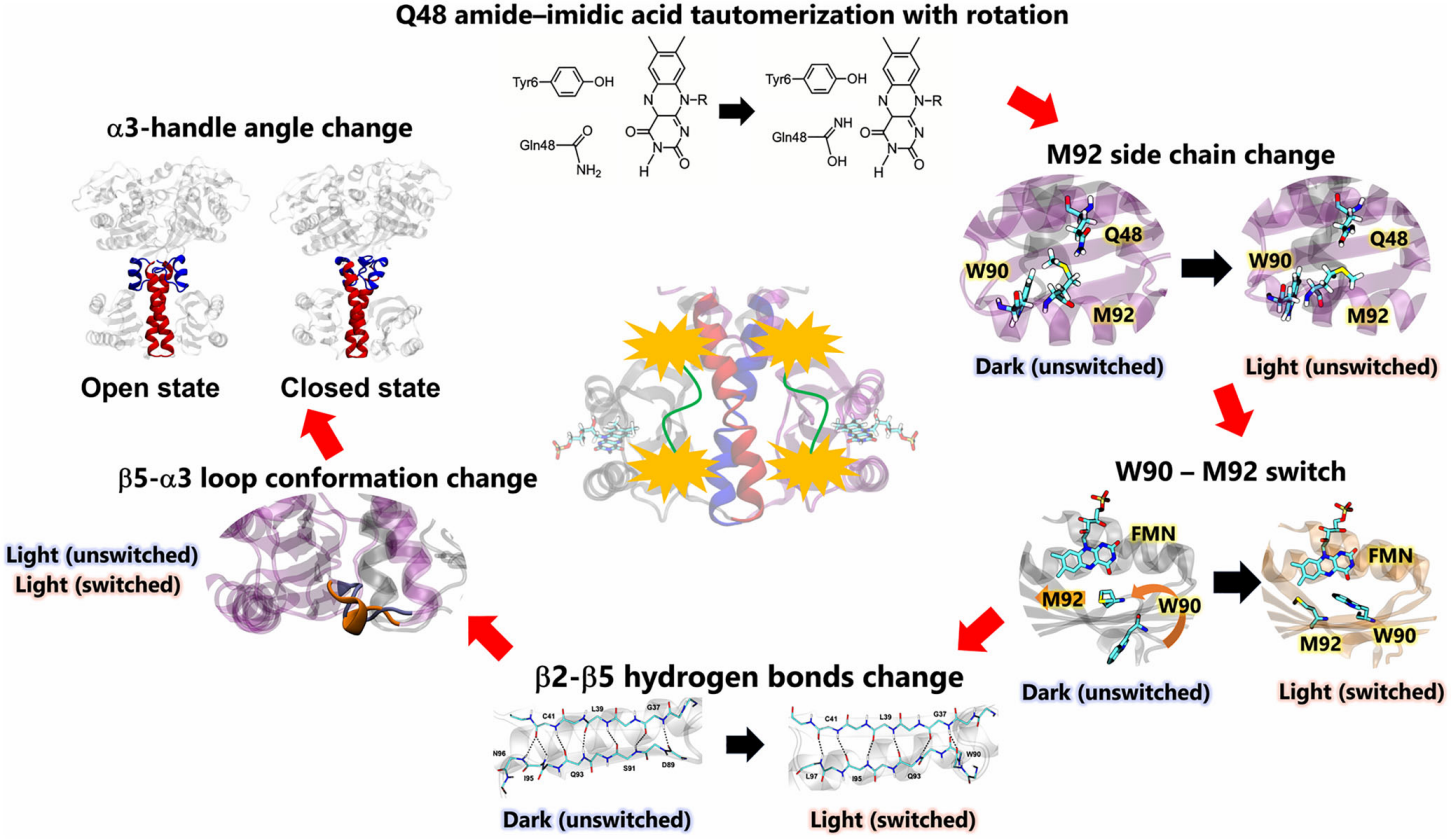
Proposed mechanism of the light‐induced allosteric signal transduction in OaPAC. The schematic illustrates the sequential structural transitions from the initial photoactivation in the blue‐light‐using flavin domain to the global conformational changes, progressing clockwise from the top. (1) Blue‐light irradiation triggers amide‐imidic acid tautomerization and rotation of the Q48 side chain. (2) This local rearrangement of the hydrogen‐bonding network induces a conformational shift in the M92 side chain. (3) The inward movement of W90 characterizes the subsequent W90–M92 switch. (4) This major structural switch drives the sliding of the β5‐sheet, altering the main‐chain hydrogen bonding between the β2‐ and β5‐sheets. (5) Consequently, the β5–α3 linker loop forms a helix. (6) Ultimately, these dynamic shifts propagate to the dimer interface, inducing a change in the angle between the central α3‐helices and the handle region, which drives the transition from the open to the closed conformation required for adenylyl cyclase activation.

Our structural insights, based on MD simulations, provide a foundation for understanding the mechanisms required to optimize PACs as optogenetic tools. However, several energetic and functional aspects of these transitions remain to be elucidated. Future work should focus on using the W90–M92 transition as a reaction coordinate for umbrella sampling, thereby enabling a rigorous discussion of the free‐energy landscape and the thermodynamic feasibility of this conformational switch. Furthermore, free‐energy calculations for the two distinct handle conformations are necessary to determine whether the W90–M92 switched or unswitched state is energetically more favorable under specific conditions. To clarify the functional significance of the open and closed conformations, future studies should also examine whether these states are associated with changes in the geometry of the catalytic site, including the relative arrangement of catalytic residues and the ATP/Mg^2+^‐binding site. MD simulations of the apo form of OaPAC will be useful for sampling the open and closed conformational ensembles and evaluating differences in catalytic‐pocket accessibility. Subsequent ATP‐binding free‐energy calculations for these conformations will allow the relationship between the open/closed transition and substrate‐binding affinity to be quantitatively assessed. Such analyses will be important for establishing how the conformational changes identified in the present study are coupled to catalytic activation and for guiding the rational design of high‐performance PAC‐based optogenetic actuators.

## 4 MATERIALS AND METHODS

### 4.1 Preparation of simulation model

Three initial simulation models—the WT dark state, the unswitched WT light state, and the unswitched L111A/L115A light state—were constructed based on the x‐ray crystal structure of OaPAC in the dark state (PDB ID: 4YUS; Ohki et al., 2016), and the initial simulation model of the W90–M92 switched WT light state was constructed based on the X‐ray crystal structure of OaPAC in the W90–M92 switched light state (PDB ID: 8QFG; Chretien et al., 2024) (Figure S13). Since the PDB entries provide only monomeric structures, OaPAC homodimers were generated using (Schrödinger, n.d.) (https://pymol.org/). Missing C‐terminal residues (residues 351–366) were complemented using Modeller (Sali & Blundell, 1993). The protonation states of histidine residues (H70, H119, and H345) were determined from their local environments, resulting in assignments of HIE, HID, and HID, respectively. The protonation states of aspartate and glutamate residues were assumed to be deprotonated. Because the 4YUS crystal structure lacks electron density for the substrate analog (ApCpp) and the magnesium ions, the substrate ATP and magnesium ions were modeled by superimposing those from the bPAC structure (PDB ID: 5MBK; Lindner et al., 2017) using ProFit (McLachlan, 1982; Martin, n.d.).

System preparation was performed using the LEaP module of Amber20 (Case et al., 2020). The AMBER ff14SB (Maier et al., 2015) force field was employed for the protein. Force field parameters of FMN were taken from our previous study (Taguchi et al., 2024 The TIP3P model (Jorgensen et al., 1983) was used for water molecules. Force field parameters of ATP were obtained from the AMBER parameter database (Meagher et al., 2003). The protein was solvated with a minimum distance of 18.0 Å to the boundary of the simulation unit box, and 150 mM NaCl was added to mimic physiological conditions.

### 4.2 Molecular dynamics simulations

MD simulations were performed on four states: WT dark, unswitched WT light, unswitched L111A/L115A light, and switched WT light. Energy minimization, heating, and equilibration were conducted using Amber20 (Case et al., 2020). A key methodological feature of this study is the treatment of each photoreceptor region (corresponding to the QM region in the previous study, Taguchi et al., 2024) as an independent rigid body during conformational sampling (Figure S14). After equilibration simulation, the atomic charges and coordinates of the photoreceptor regions were updated to the values obtained from QM/MM free‐energy geometry optimization (Taguchi et al., 2024).

#### 4.2.1 WT dark, W90–M92 unswitched WT light, and unswitched L111A/L115A light states

First, energy minimization was performed for 10,000 cycles with a 12 Å cutoff and a 3.0 kcal/(mol·Å^2^) restraint on the Cα atoms. The systems were then heated to 300 K over 300 ps using Langevin dynamics in the constant number of particles, volume, and temperature (NVT) ensemble with a 2 fs time step and a 10 Å cutoff, maintaining the 3.0 kcal/(mol·Å^2^) Cα restraint. Subsequently, a 5 ns pre‐equilibration simulation in the constant number of particles, pressure, and temperature (NPT) ensemble was conducted, gradually reducing the Cα restraints (3.0, 1.0, 0.5, 0.2, and 0.1 kcal/(mol·Å^2^)) for every 1 ns. Final equilibration simulation in the NPT ensemble was performed without Cα restraints using Langevin dynamics. Then, the atomic coordinates and charges in each photoreceptor region were updated to incorporate the QM/MM‐optimized parameters, making the WT dark, unswitched light, and L111A/L115A light models. Conformational sampling was then performed in the NVT ensemble using an in‐house modified version of Amber20 that treats each photoreceptor region as a rigid body (dubbed “multi‐rigid‐body” code). For each model, five independent 3 μs production runs starting from different initial velocities were executed with a 2 fs time step and a 10 Å cutoff.

#### 4.2.2 W90–M92 switched WT light state

Note that for the W90–M92 switched WT light‐state model, the atomic charges in each photoreceptor region were updated to QM/MM‐optimized values before energy minimization, as the starting structure (PDB ID: 8QFG; Chretien et al., 2024) already represents the light state. Energy minimization, heating, and equilibration were performed following the same protocol described above. Before production sampling, only the QM region coordinates were updated to the QM/MM‐optimized geometry. The sampling duration and simulation conditions were the same as those used for the other models.

### 4.3 Analysis of molecular dynamics simulations

MD trajectories were analyzed using the CPPTRAJ module in Amber20 (Case et al., 2020) and the MDTraj Python library (McGibbon et al., 2015).

All statistical plots were generated using the *matplotlib* library in Python (https://matplotlib.org).

#### 4.3.1 Root‐mean‐square fluctuation

The RMSF of the backbone atoms (C, N, O, and Cα) was calculated for residues 1–350. The highly flexible C‐terminal region (residues 351–366) was excluded from the analysis due to its large structural fluctuations. The resulting RMSF profiles were obtained by averaging the values between monomers A and B (Figure 2b).

#### 4.3.2 Secondary structure

The residue‐wise average occupancy of each secondary structure element was computed (Figure S2a). To quantify structural variations relative to a reference state, the residue‐wise SSD in secondary structure occupancy was calculated using the following equation:

(1) $$\sum_{i}{\left(x_{i}^{\text{model}}-x_{i}^{\text{reference}}\right)}^{2},\;i=\text{each secondary structure},$$

where x is the average occupancy of the secondary structure, and i represents eight types of secondary structure (bend, turn, pi, alpha, 3–10, bridge, extended, and none).

#### 4.3.3 Angle between α3‐helices and handle

The angle between the α3‐helices (residues: 103–125) and the handle (residues: 127–143) was defined as illustrated in Figure 3b. Because the angles sampled across the MD trajectories ranged approximately from 0° to 80°, the midpoint of this range (40°) was adopted as an operational cutoff. Based on this cutoff, closed and open conformational states were defined by angles below and above 40°, respectively.

#### 4.3.4 Difference residue‐wise hydrogen‐bonding analysis

Residue‐wise hydrogen‐bonding frequencies were determined using the Baker–Hubbard identification method (Baker & Hubbard, 1984). The geometric criteria for a stable hydrogen bond were defined by an acceptor–hydrogen distance cutoff of 3.0 Å and a donor–hydrogen–acceptor angle threshold of 135°. To focus on non‐local interactions and eliminate trivial backbone constraints, adjacent contiguous residue pairs (i and i+1) were excluded from the analysis. Differences in hydrogen‐bonding frequencies were subsequently evaluated by subtracting the reference model occupancies from those of each target model.

#### 4.3.5 Contact frequency

Residue‐wise difference contact frequencies were evaluated using an all‐atom distance cutoff of 4.5 Å. A contact was defined to exist if any atom of a given residue came within this threshold distance of any atom belonging to another residue. Differences in contact frequency were calculated across the relevant simulation trajectories to map localized gains or losses in packing interfaces.

#### 4.3.6 Principal component analysis

PCA was performed on the backbone atoms (C, N, O, and Cα), using the WT dark state after equilibration as the reference structure. To compare principal modes among four models directly, the eigenvalues of each model were scaled to the W90–M92 switched WT light‐state model as the reference model using the following equation:

(2) $$\begin{array}{c} \Lambda^{\prime }=\Sigma \times \Lambda =\left(\begin{array}{ccc} \sigma_{11} & \sigma_{12} & \sigma_{13}\\ \sigma_{21} & \sigma_{22} & \sigma_{23}\\ \sigma_{31} & \sigma_{32} & \sigma_{33} \end{array}\right)\left(\begin{array}{c} \lambda_{1}\\ \lambda_{2}\\ \lambda_{3} \end{array}\right) \end{array},$$

$$\sigma_{\mathrm{ij}}=v_{i,\text{model}}^{T}\times v_{j,\text{reference}},$$

where $\Lambda^{\prime }$ is the eigenvalue matrix after the scaling, Σ is the cosine similarity matrix between the target model and the reference one, v is the eigenvector, and indexes *i, j* are the number of the considered eigenvector. In this study, only the top three principal modes (*i* = 1, 2, 3) were retained for extensive analysis, as the remaining modes contributed negligibly to the overall structural variance.

The percentage contribution of each individual mode presented in Table 2 was calculated using the following equation:

(3) $$\begin{array}{c} {\text{Contribution}}_{i,\text{model}}=\frac{\lambda_{i,\text{model}}}{\sum_{i}\lambda_{i,\text{model}}} \end{array}.$$

## AUTHOR CONTRIBUTIONS

**Akiya Fukuda**: Conceptualization; data curation; formal analysis; investigation; methodology; software; validation; visualization; writing—original draft preparation. **Masahiko Taguchi**: Conceptualization; funding acquisition; investigation; methodology; writing—review and editing; resources; supervision; software; validation. **Justin Chan**: Writing—review and editing. **Shun Sakuraba**: Conceptualization; methodology; writing—review and editing; supervision. **Eriko Nango**: resources; writing—review and editing. **Hidetoshi Kono**: Conceptualization; methodology; funding acquisition; project administration; supervision; writing—review and editing.

## CONFLICT OF INTEREST STATEMENT

The authors declare no conflicts of interest.

## Supporting information


**Movie S1.** Flexion (corresponding to *λ*_1_).

**Movie S2.** Twisting of the BLUF‐AC domains (corresponding to *λ*_2_).

**Movie S3.** Opening of the AC domains (corresponding to *λ*_3_).

**Data S1.** Supporting Information.

## Data Availability

The trajectory for all states, the Python scripts for analysis, and all results are available on Zenodo (https://doi.org/10.5281/zenodo.21211894) and GitHub (https://github.com/Fukuda-akiya/oapac-hybridMD). See Section 4 for detailed descriptions of the software, along with workflows and parameter settings of the simulations. Coordinate data of the optimized structures for Y6, Q48, and FMN by QM/MM reweighting free‐energy self‐consistent field calculations are available on https://doi.org/10.1021/acsphyschemau.4c00040.
