# Supplementary material for: Structural insights into signal transduction in photoactivated adenylyl cyclase through molecular dynamics simulations
Source: Protein Sci. 2026 Nov 5;35(12):e70830. doi: 10.1002/pro.70830 (PMC13740344; doi:10.1002/pro.70830)
Supplement: Supplementary file 1 [file PRO-35-e70830-s001.pdf]

Supplementary material

Structural Insights into Signal Transduction in Photoactivated Adenylyl Cyclase through Molecular Dynamics Simulations

Akiya Fukuda[1,+], Masahiko Taguchi[1,2,+*], Justin Chan[3], Shun Sakuraba[3,4], Eriko Nango[1,2,5], and Hidetoshi Kono[3,4*]

[1] *Graduate School of Science, Tohoku University, Sendai 980-0845, Japan*

[2] *Institute of Multidisciplinary Research for Advanced Materials, Tohoku University, Sendai, Miyagi, 980-8577, Japan*

[3] *Institute for Quantum Life Science, National Institutes for Quantum Science and Technology, Chiba 263-8555, Japan*

[4] *Center of Quantum Life Science for Structural Therapeutics, Chiba University, Chiba 263-8522, Japan*

[5] *International Center for Synchrotron Radiation Innovation Smart, Tohoku University, Sendai, Miyagi, 980-8572, Japan*

E-mail: kono.hidetoshi@qst.go.jp, Phone: +81-43-382-4295

+equally contributed

*** corresponding authors**

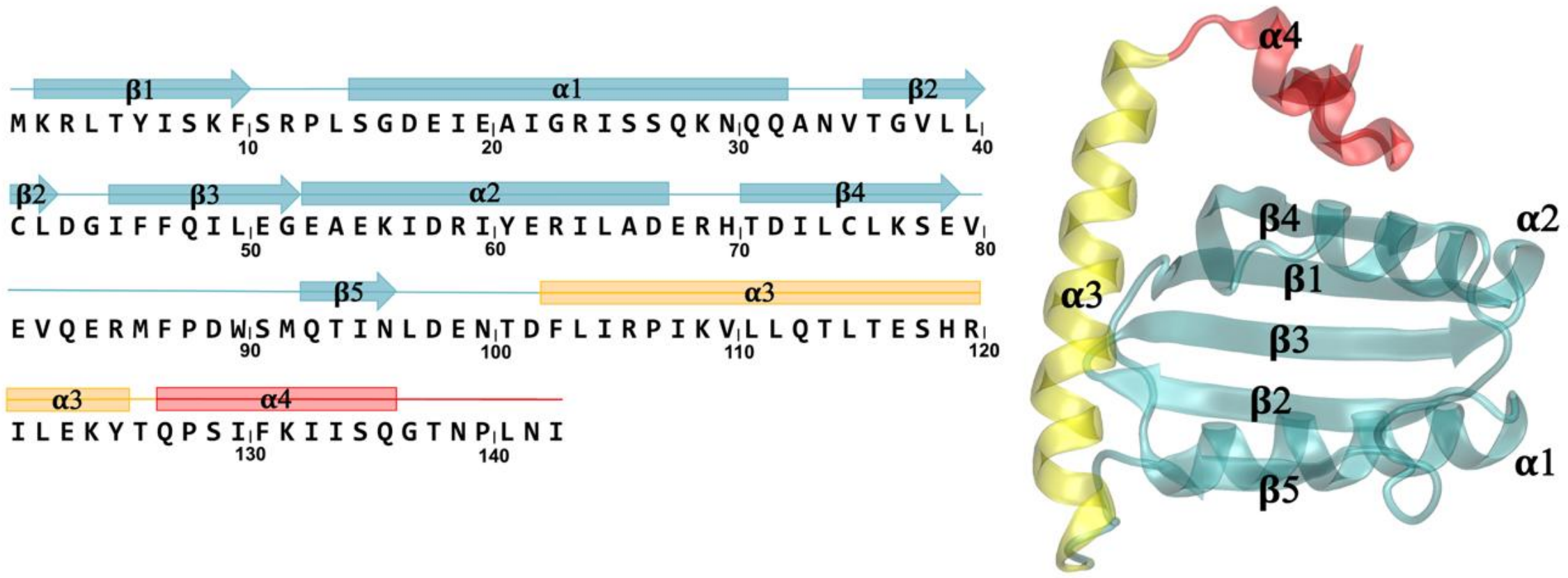

FIGURE S1. The amino acid sequence and secondary structures of the BLUF domain (residues 1–143) of OaPAC in the dark state (PDB ID: 4YUS [1]).

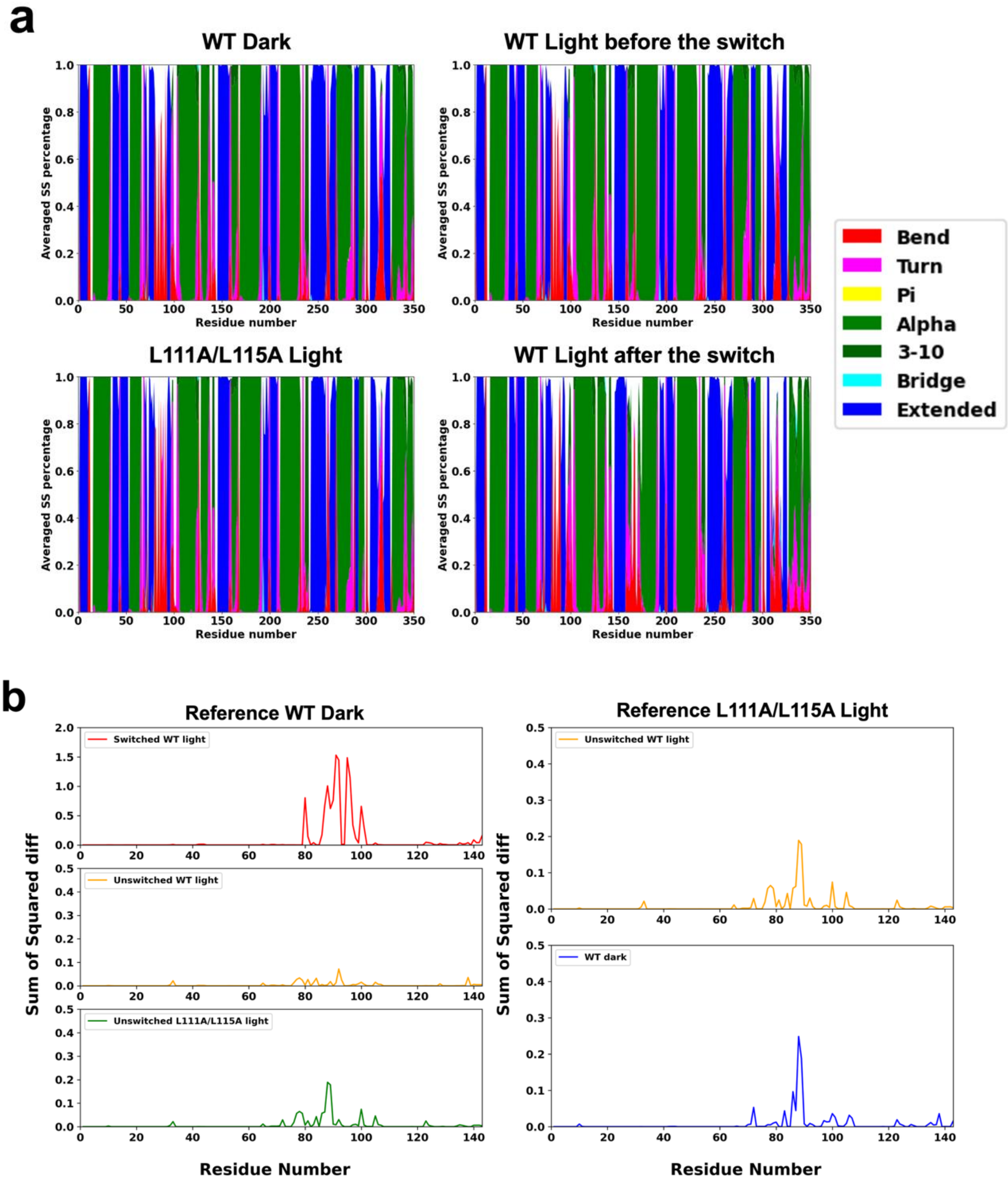

FIGURE S2. Averaged secondary structure occupancy and the sum of squared differences (SSD) between models. **a** Average occupancy of secondary structure in each model from the MD trajectory. The colors correspond to each secondary structure. **b** Sum of squared differences ($\sum_i \left(x_i^{\text{model}} - x_i^{\text{reference}}\right)^2$, i = each secondary structure) between two models. In the left panel, the differences from the WT dark state are shown for the unswitched WT light (orange), switched WT light (red), and L111A/L115A light (green) states. In the right panel, the differences from the L111A/L115A light state are shown for the WT dark (blue) and the unswitched WT light (orange) states.

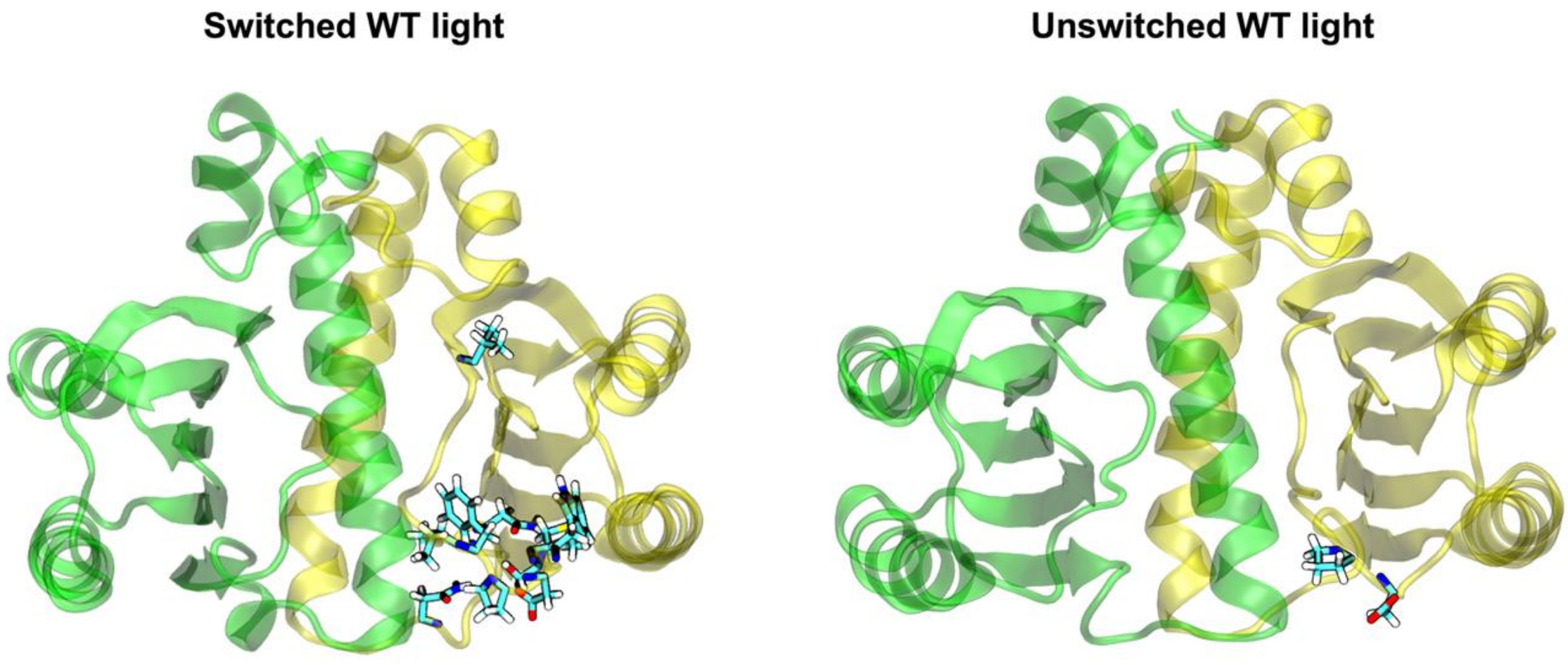

FIGURE S3. Mapping of the residues onto the representative structure from the MD simulation trajectory. Right: Switched WT light state. The residues whose sum of squared differences (SSD) is greater than 0.5, based on the WT dark state as a reference, are shown. Left: Unswitched WT light state. The residues whose SSD is greater than 0.1, based on the L111A/L115A light state as a reference, are shown.

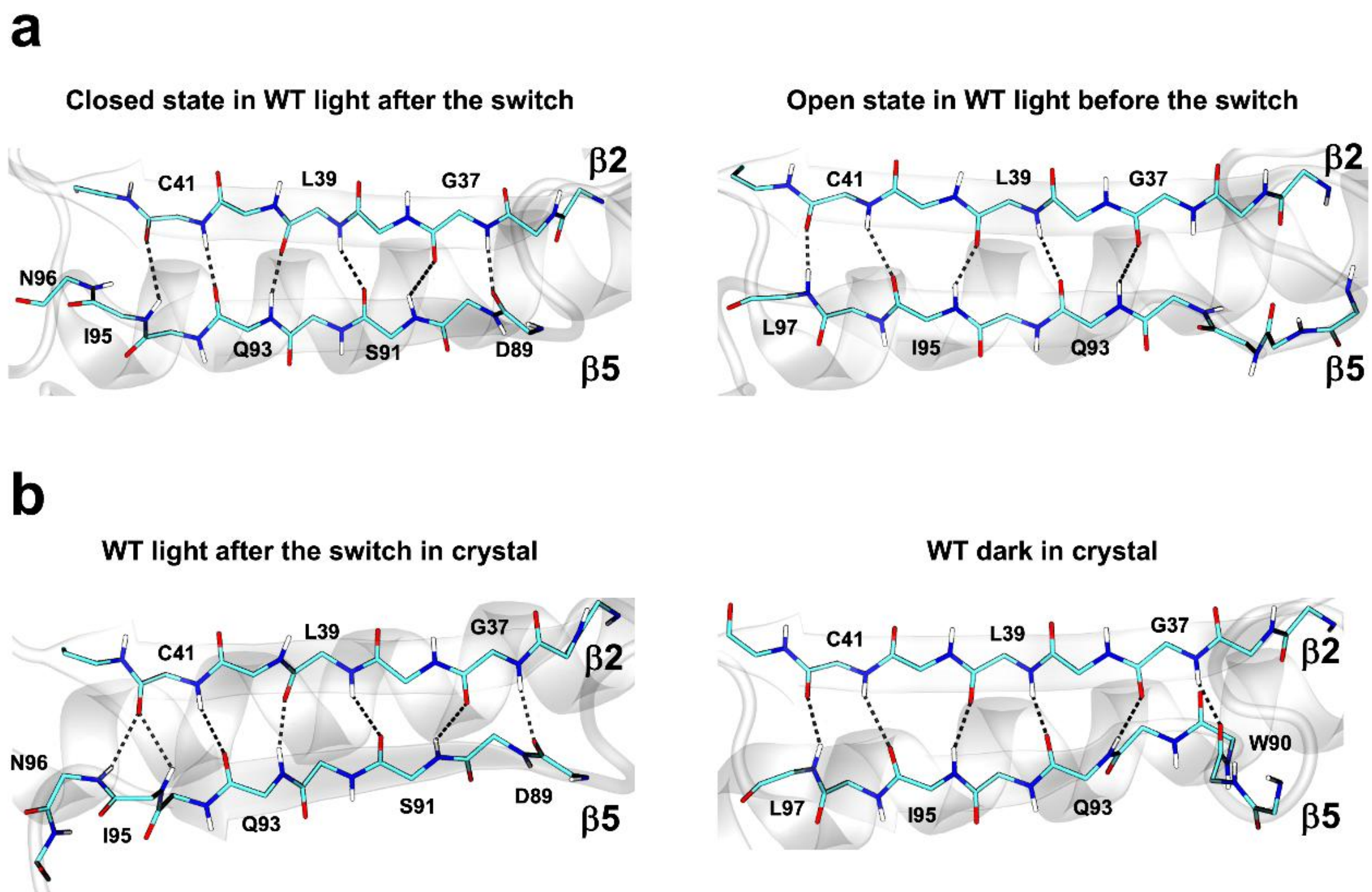

FIGURE S4. Comparison of the hydrogen-bonding pattern between the β2- and β5-sheets. a Representative structures from the MD simulation trajectories. b Crystallographic structures: the left is the WT dark state (PDB ID: 4YUS; Ohki et al., 2016) and the right is the W90–M92 switched WT light state (PDB ID: 8QFG; Chretien et al., 2024).

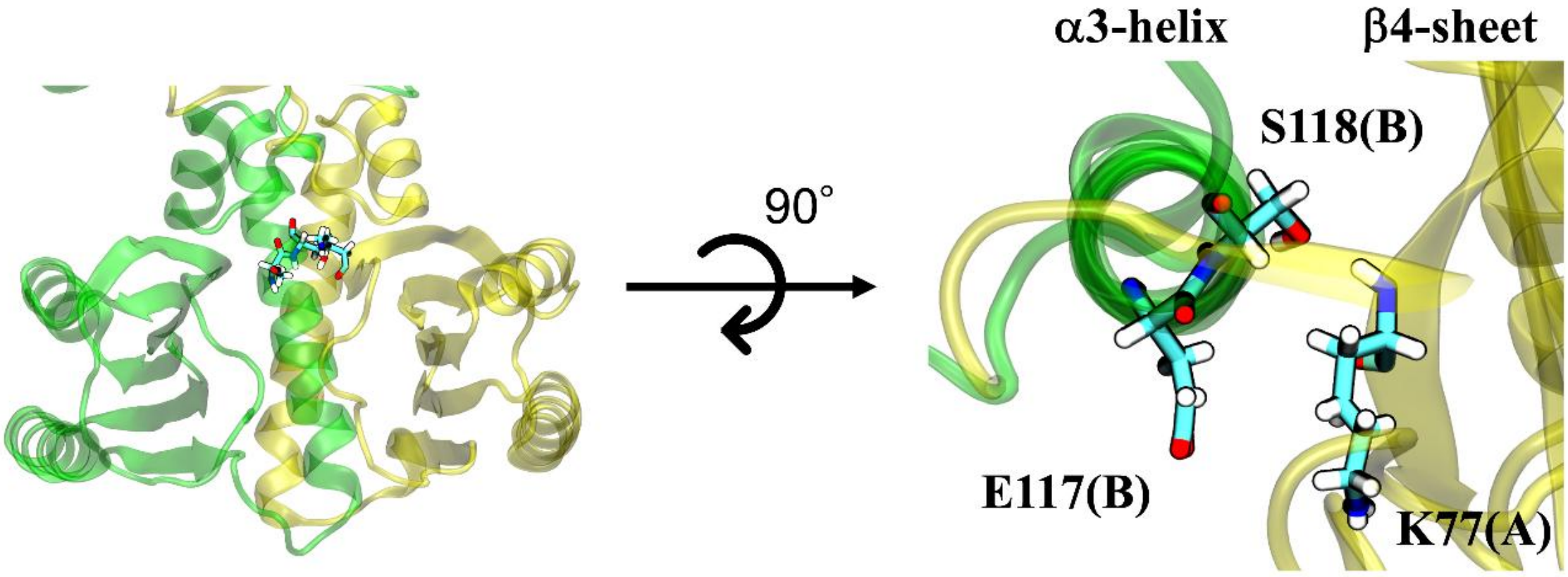

K77(A) – S118(B): +0.25
K77(A) – E117(B): -0.03

FIGURE S5. Difference in inter-monomer hydrogen bond frequencies between the unswitched WT light and WT dark states for K77(A)–S118(B) and K77(A)–E117(B). Values indicate the change in hydrogen bond frequency (unswitched WT light − WT dark). The structure shown is a representative snapshot from the unswitched WT light trajectory, with the right panel showing a 90-degree-rotated view highlighting the spatial arrangement of the

a

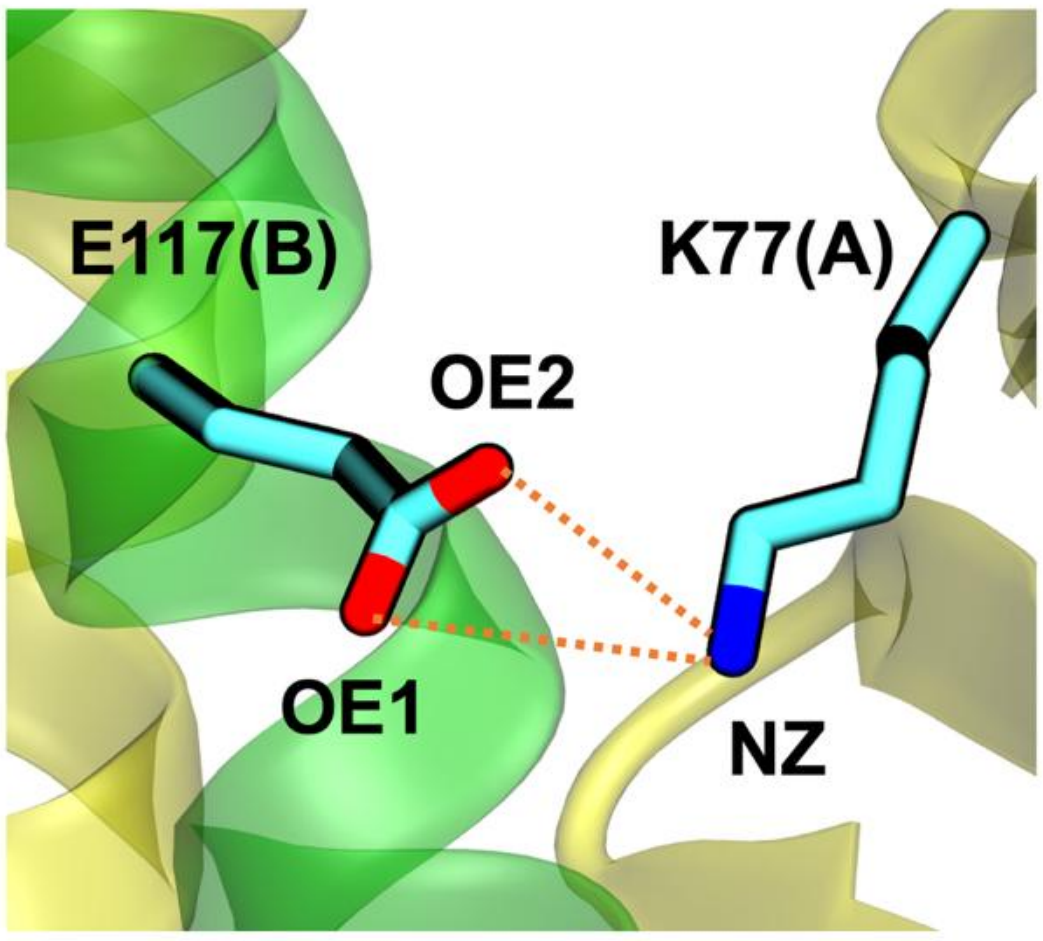
E117(B)
K77(A)
OE2
OE1
NZ

b

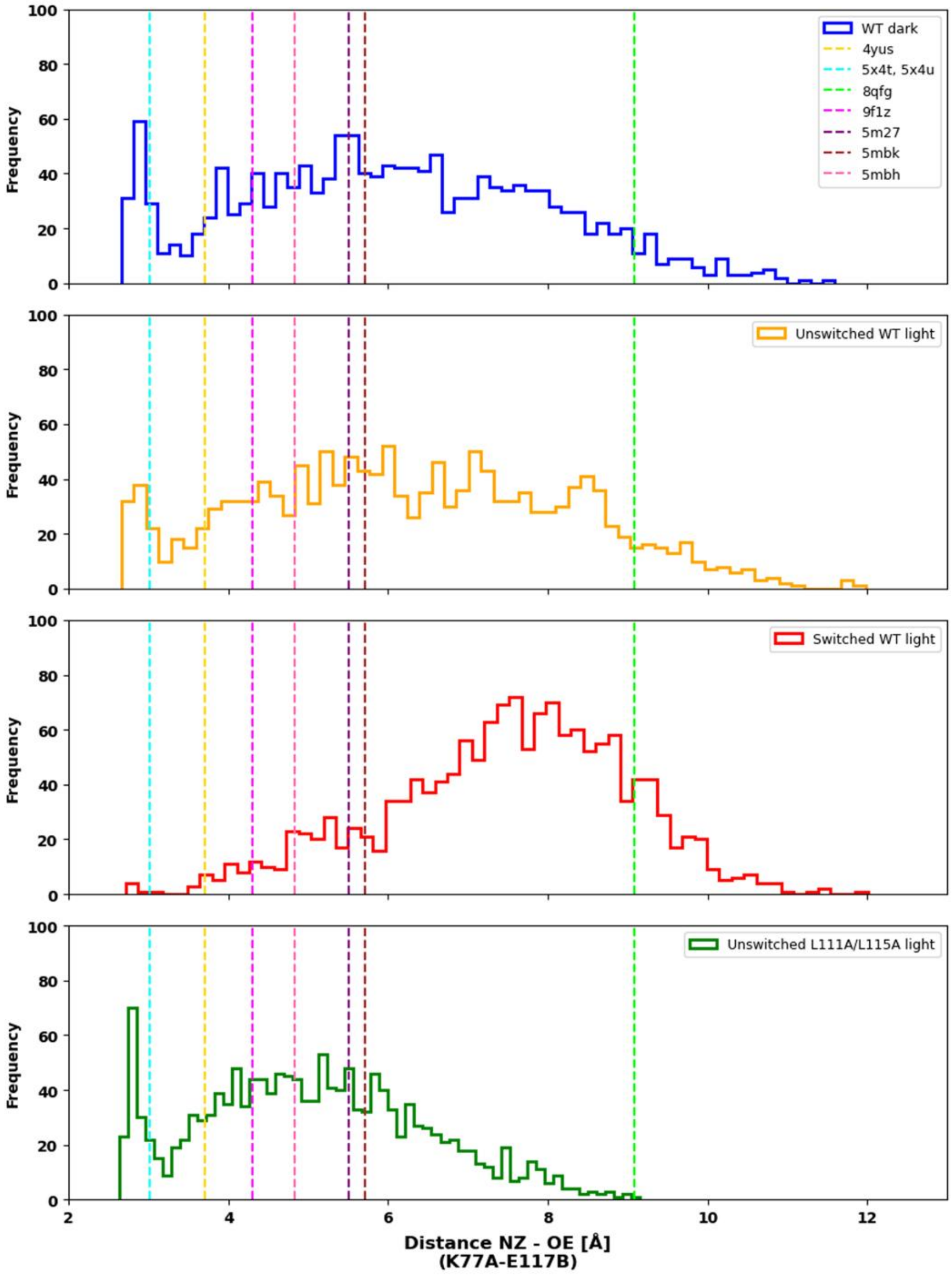
WT dark
4yus
5x4t, 5x4u
8qfg
9f1z
5m27
5mbk
5mbh
Unswitched WT light
Switched WT light
Unswitched L111A/L115A light
Frequency
Distance NZ - OE [Å]
(K77A-E117B)

FIGURE S6. Analysis of the inter-monomer hydrogen bond between K77(A) and E117(B) **a** The definition of the interaction distance (PDB ID: 4YUS; Ohki et al., 2016). **b** The distance distribution in four models: WT dark (*blue*), unswitched WT light (*orange*), switched WT light (*red*), and unswitched L111A/L115A light (*green*). Dashed lines are the distances of the crystallographic structures; *Yellow* is OaPAC WT dark (PDB ID: 4YUS; Ohki et al., 2016), *cyan* are OaPAC WT light at 20 s (PDB ID: 5X4T) and OaPAC WT light at 60 s (PDB ID: 5X4U; Ohki et al., 2017), *lime* is OaPAC W90-M92 switched WT light (PDB ID: 8QFG; Chretien et al., 2024), *magenta* is Y6W OaPAC dark (PDB ID: 9F1Z; Kapetanaki et al., 2024), *purple* is bPAC WT dark (PDB ID: 5M27; Lindner et al., 2017), *brown* is Y7F bPAC quasi-light (PDB ID: 5MBK; Lindner et al., 2017), and *pink* is Y126F/N257A bPAC quasi-light (PDB ID: 5MBH; Lindner et al., 2017). The x-axis is the distance between the NZ of K77 and OE1 or OE2 of E117.

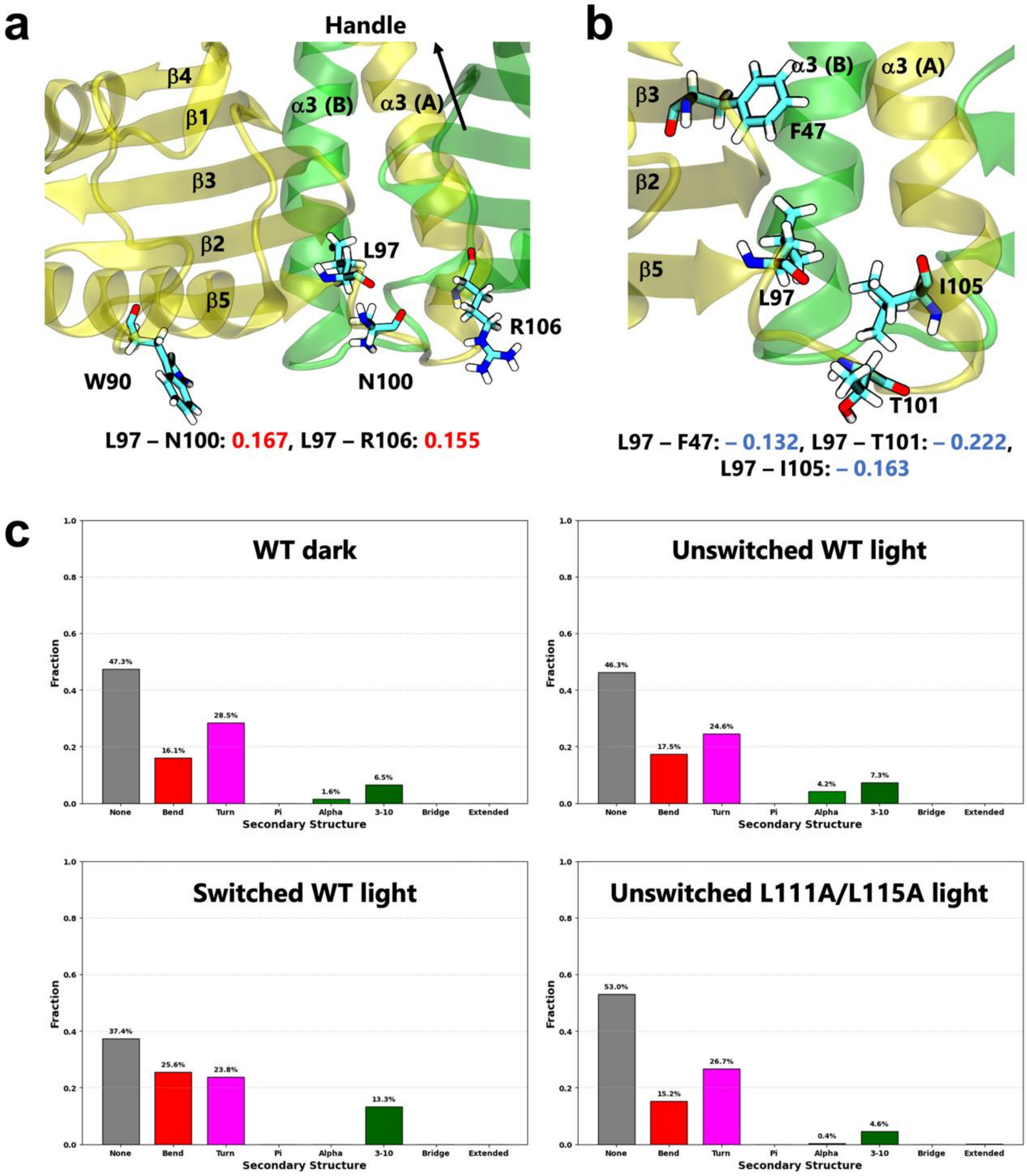

FIGURE S7. Contact-frequency changes of L97. a Mapping of the residues with increased contact frequency onto the representative structure of the unswitched WT light state. b Mapping of the residues with decreased contact frequency onto the representative structure of the unswitched WT light state. c The residue-averaged secondary structure occupancy for the linker between the β5-sheet and α3-helix (residues 97–102) in the four models.

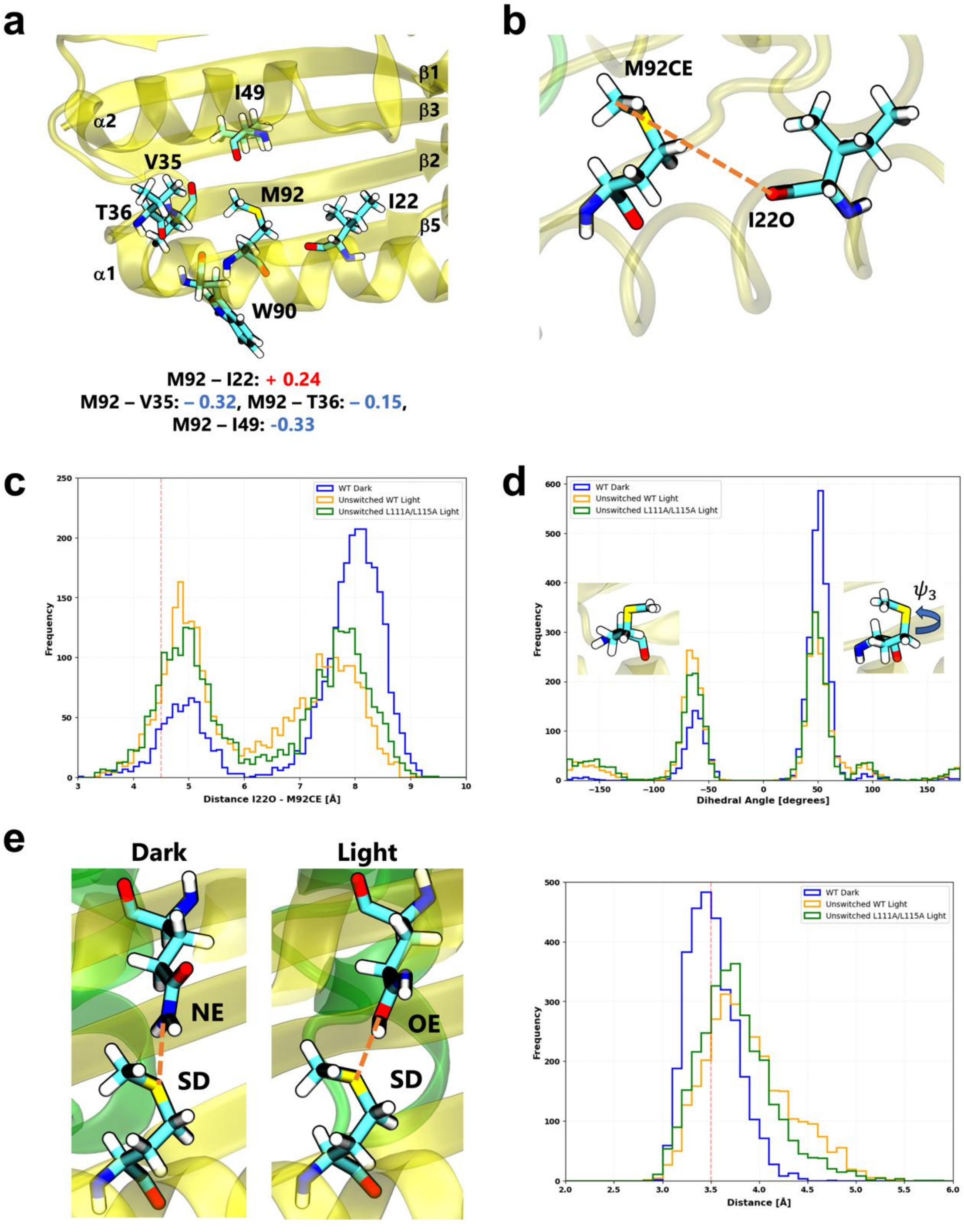
a
b
c
d
e
β1
β3
α2
I49
V35
β2
M92
I22
T36
β5
α1
W90
M92 – I22: + 0.24
M92 – V35: – 0.32, M92 – T36: – 0.15,
M92 – I49: -0.33
M92CE
I22O
WT Dark
Unswitched WT Light
Unswitched L111A/L115A Light
Frequency
Distance I22O - M92CE [Å]
Dihedral Angle [degrees]
ψ_3
Dark
Light
NE
SD
OE
SD
Distance [Å]

FIGURE S8. M92 interaction changes. **a** Mapping of the residues showing contact-frequency changes with M92 onto the representative structure of the unswitched WT light state. **b** The definition of the distance between I22 and M92 for computation. **c** The distance distribution of I22O–M92CE for the WT dark, unswitched WT light, and unswitched L111A/L115A light states. The red dashed line is the van der Waals cutoff, 4.5 Å. **d** The dihedral angle distribution of M92 for the WT dark, unswitched WT light, and unswitched L111A/L115A light states. An angle of nearly 50° corresponds to the methyl group oriented opposite to I22, whereas an angle of around −75° corresponds to the methyl group oriented toward I22. **e** The definition of the distance between Q48 and M92 in the dark and light states, and its distribution for the WT dark, unswitched WT light, and unswitched L111A/L115A light states. The red dashed line is the van der Waals cutoff, 4.5 Å.

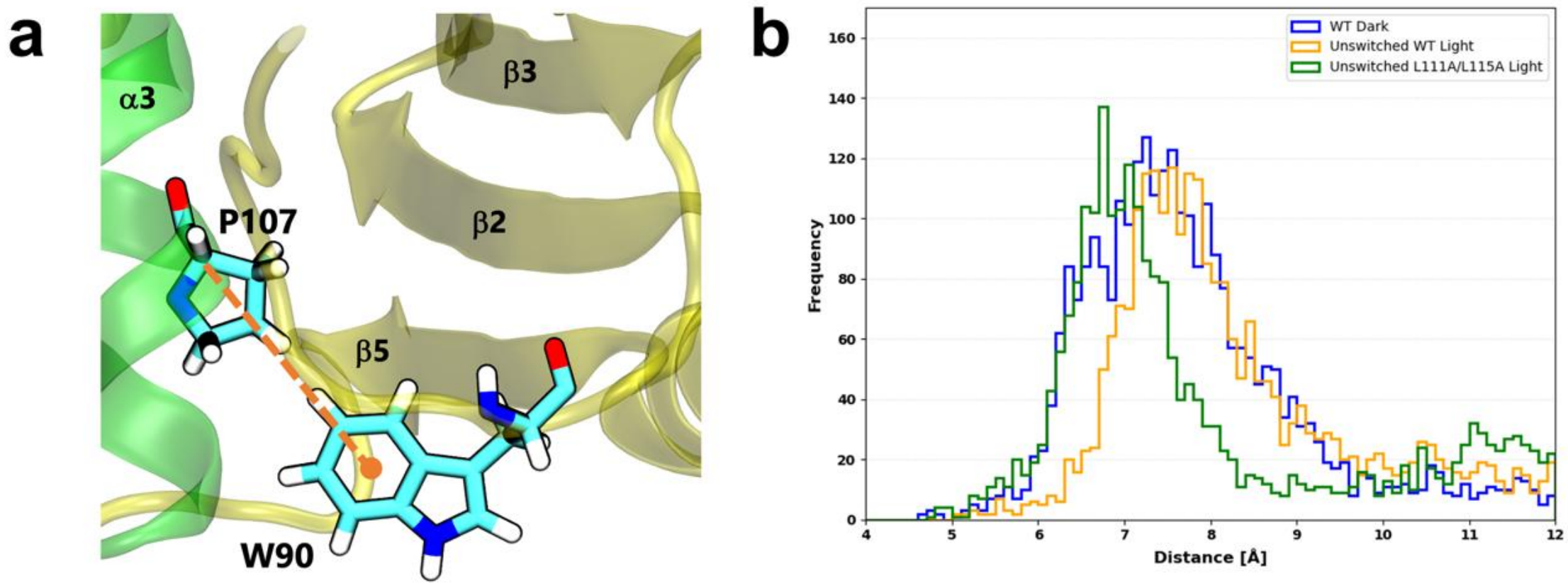

FIGURE S9. Distance between W90(A) and P107(B). **a** The definition of the distance between the centroid of W90 and the Cα atom of P107, shown on the representative structure of the WT dark state. **b** The distribution of these distances. Blue, red, and green correspond to the WT dark, unswitched WT light, and unswitched L111A/L115A light states, respectively.

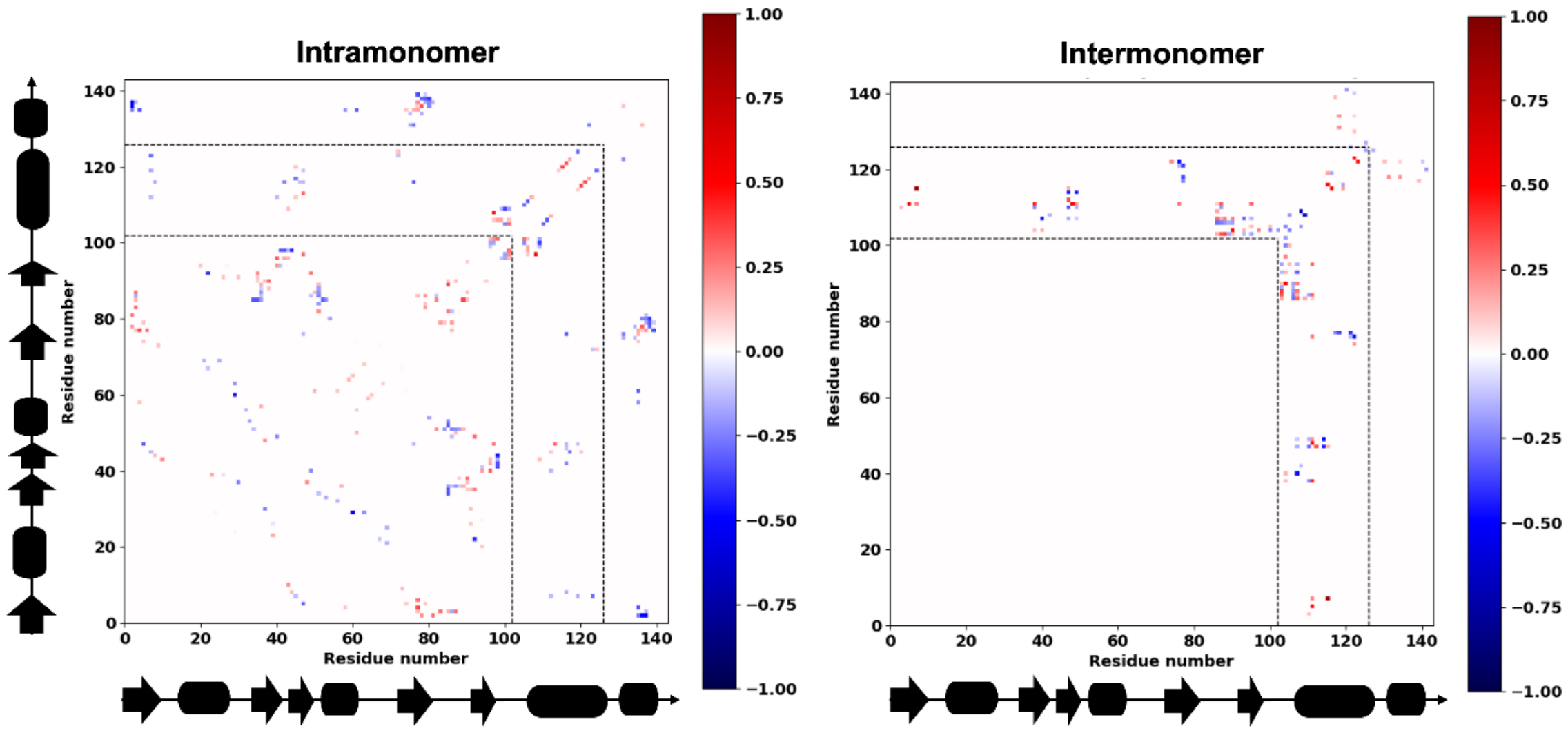

FIGURE S10. Difference contact map between the WT dark and the unswitched L111A/L115A light states. Red indicates the contacts in the WT dark state, and blue indicates those in the L111A/L115A light state. Arrows and cylinders denote the β-sheets and α-helices, respectively.

a

Intermonomer interaction of L111

Ranking	Residue pair	Ratio
1	T5 – L111	0.525
2	Q48 – L111	0.525
3	V38 – L111	0.429
4	F87 – L111	0.317
5	I95 – L111	0.297
6	L76– L111	0.268
7	I7 – L111	0.248
8	I49 – L111	0.207
9	M86 – L111	0.101

b

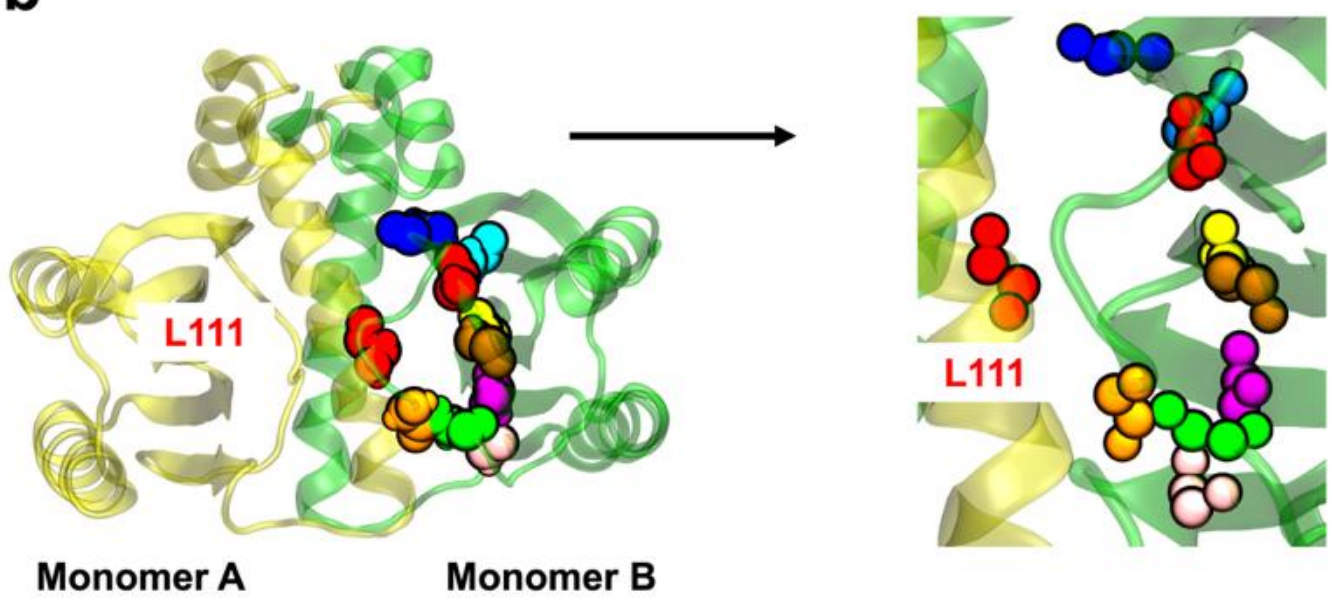

c

Intermonomer interaction of L115

Ranking	Residue pair	Ratio
1	I7 – L115	0.869
2	L115 – T116	0.479
3	L115 – H119	0.190
4	F47 – L115	0.153

d

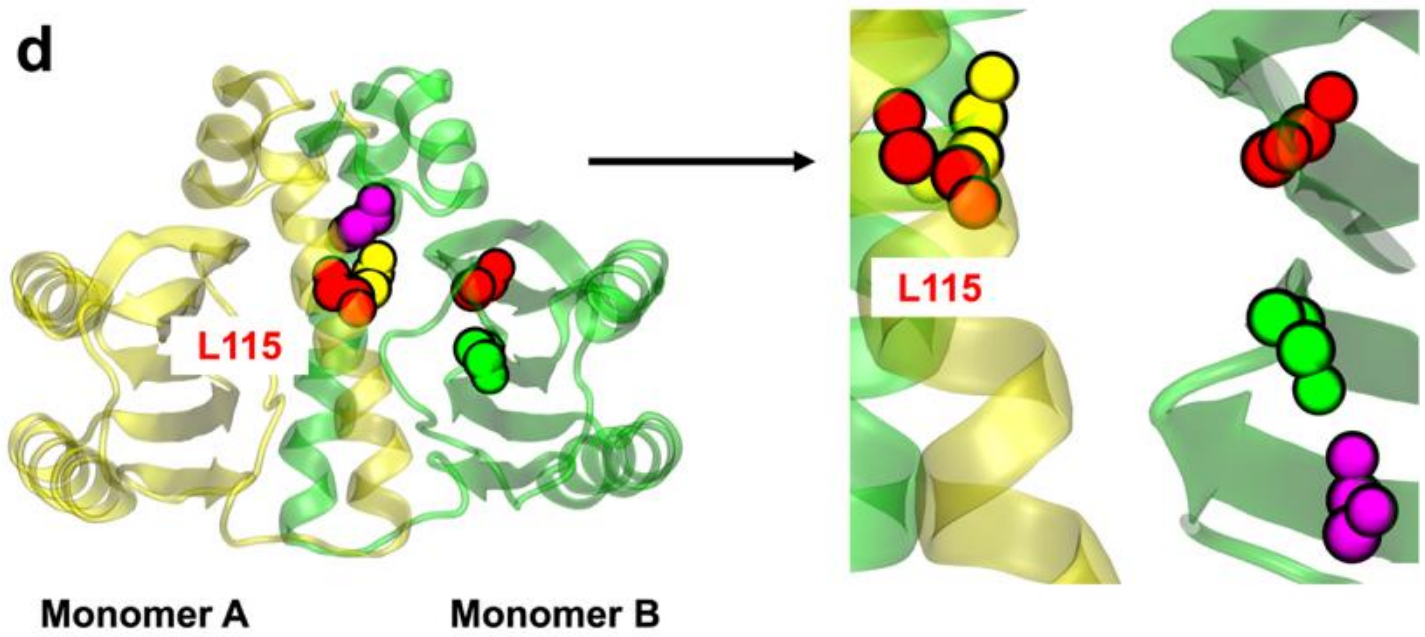

FIGURE S11. Analysis of residue-wise intermonomer contacts focused on residues L111 and L115 in the WT dark and L111A/L115A light states. **a** A ranking of the difference in contact frequency of L111 between the WT dark and L111A/L115A light states. **b** The WT dark model averaged over the 5 μs trajectory. The protein backbone is drawn in a ribbon representation, and the backbone atoms of the residues listed in **a** are depicted in the van der Waals model. **c** A ranking of the difference in contact frequency of L115 between the WT dark and L111A/L115A light states. **d** The backbone atoms of the residues listed in **c** are depicted in the van der Waals model.

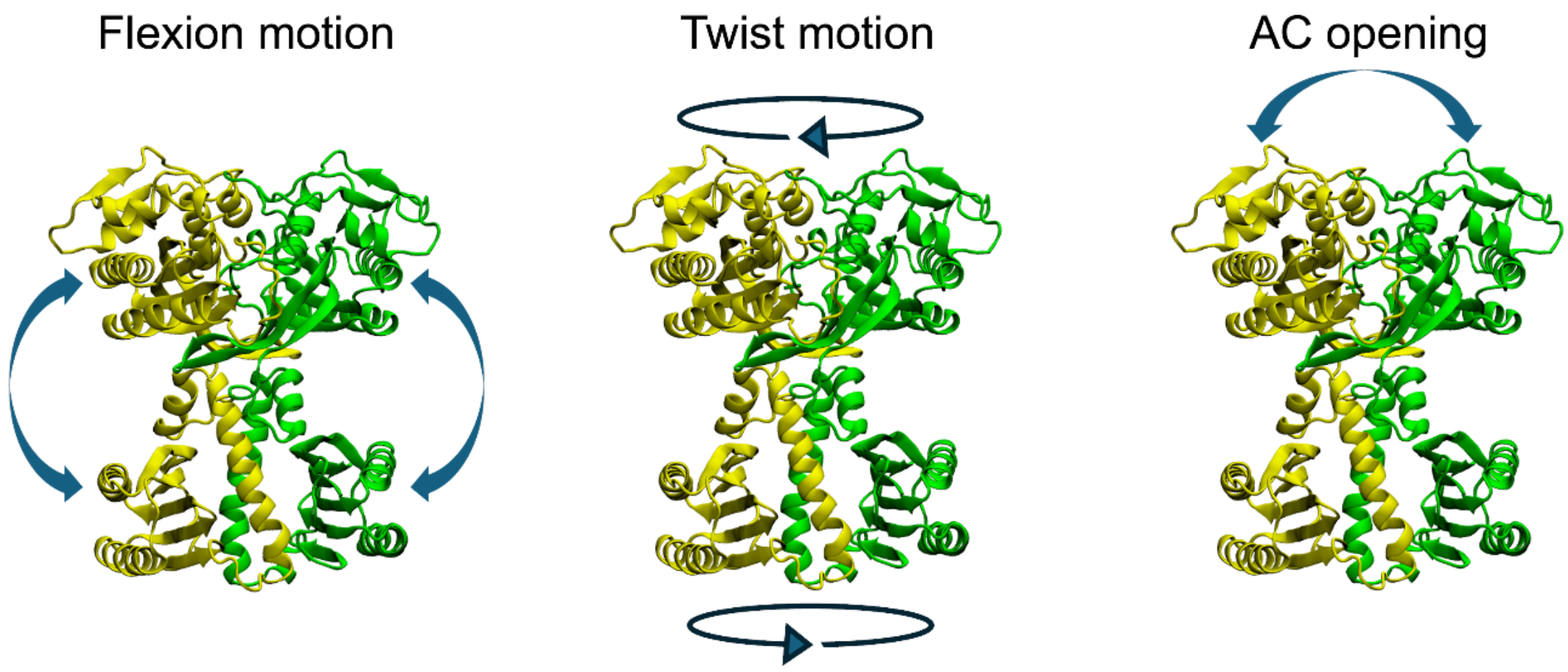

Figure S12. Mode component for each model by principal component analysis (PCA). Motion for each mode: Mode 1 is the flexion motion, mode 2 is the twist motion between the BLUF and the AC domain, and mode 3 is the open motion of the AC domain.

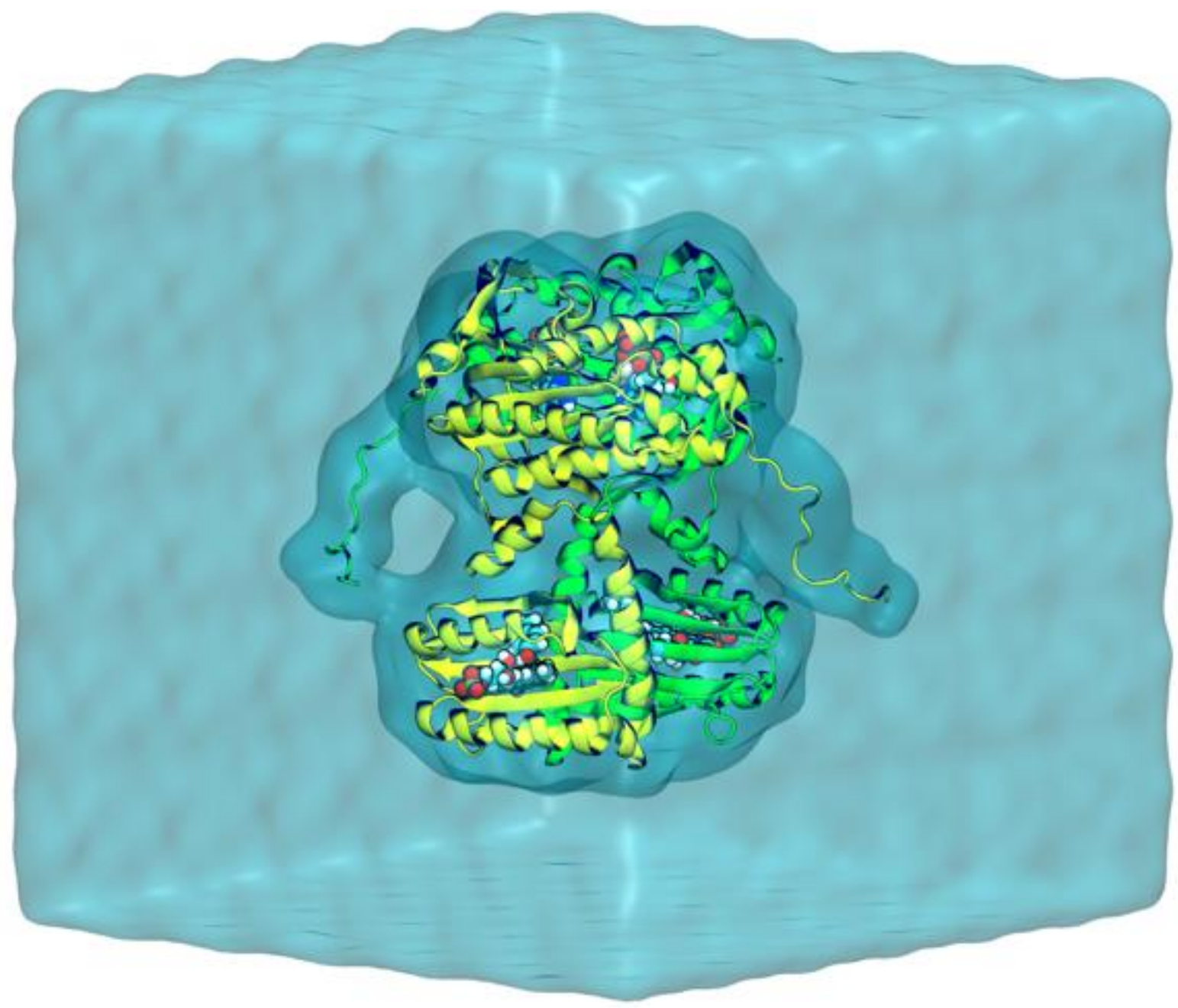

FIGURE S13. MD simulation system of OaPAC in the dark state under periodic boundary conditions. FMN, Y6, Q48, ATP, and Mg^{2+} are shown in a van der Waals representation.

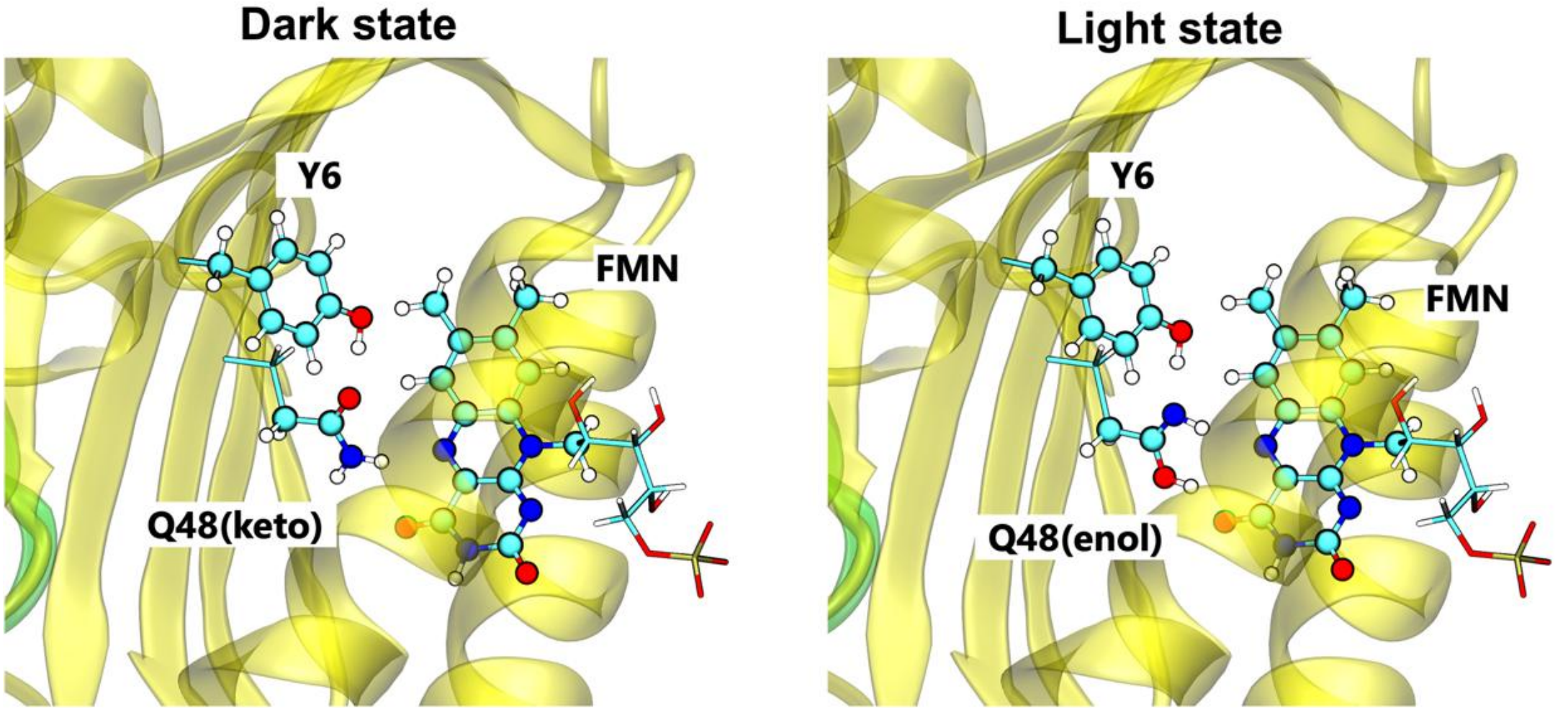

FIGURE S14. QM atoms of Y6, Q48, and FMN. The atoms shown by ball and stick representation are the QM region (53 atoms), and the all other region including stick representation of FMN is the MM region, respectively (Taguchi et al., 2024).

REFERENCES

1. Ohki, M.; Sugiyama, K.; Kawai, F.; Tanaka, H.; Nihei, Y.; Unzai, S.; Takebe, M.; Matsunaga, S.; Adachi, S.; Shibayama, N.; Zhou, Z.; Koyama, R.; Ikegaya, T.; Takahashi, T.; Tame, J. R. H.; Iseki, M.; Park, S.-Y. Structural insight into photoactivation of an adenylate cyclase from a photosynthetic cyanobacterium. *Proc. Natl. Acad. Sci. U.S.A.* **2016**, *113*, 6659–6664.
2. Chretien, A.; Nagel, M. F.; Botha, S.; Wijn, R. d.; Brings, L.; Dömer, K.; Han, H.; Koliyadu, J. C. P.; Letrun, R.; Round, A.; Sato, T.; Schmidt, C.; Secareanu, R.-C.;Stetten, D. v.; Vakili, M.; Wrona, A.; Bean, R.; Mancuso, A.; Schulz, J.; Pearson, A. R.; Kottke, T.; Lorenzen, K.; Schubert, R. Light-induced Trp_{in}/Met_{out} Switching During BLUF Domain Activation in ATP-bound Photoactivatable Adenylate Cyclase OaPAC. *J. Mol. Biol.*, **2024**. *436*. 168439-168456.
3. Ohki M.; Sato-Tomita, A.; Matsunaga, S.; Iseki, M.; Tame, J. R. H.; Shibayama, N.; Park, S.-Y. Molecular mechanism of photoactivation of a light-regulated adenylate cyclase. *Proc. Natl. Acad. Sci. U.S.A.*, **2017**, *114*, 8562-8567.
4. Kapetanaki, S. M.; Coquelle, N.; Stetten, D. v.; Byrdrin, M.; Rios-Santacruz, R.; Bean, R.; Bielecki, J.; Boudjelida, M.; Fekete, Z.; Grime, G. W.; Hatton, C.; Kantamneni, S.; Kharitonov, K.; Kim, C.; Kloos, M.; Koua, F. H. M.; Martinez, I. d. D.; Melo, D.; Rane, L.; Round, A.; Round, E.; Sarma, A.; Schubert, R.; Schulz, J.; Sikorski, M.; Vakili, M.; Valerio, J.; Vitas, J.; Wijn, R. d.; Wrona, A.; Zala, N.; Pearson, A.; Dörner, K.; Schirò, G.; Garman, E.

F.; Lukács, A.; Weik, M. Crystal structure of a bacterial photoactivated adenylate cyclase determined by serial femtosecond and serial synchrotron crystallography. *IUCrJ*, **2024**, *11*, 991-1006.

5. Lindner, R.; Hartmann, E.; Tarnawski, M.; Winkler, A.; Frey, D.; Reinstein, J.; Meinhart, A.; Schlichting, I. Photoactivation Mechanism of a Bacterial Light-Regulated Adenylyl Cyclase. *J. Mol. Biol.* **2017**, *429*, 1336-1351.

6. Taguchi, M.; Sakuraba, S.; Chan, J.; Kono, H. Unveiling the Photoactivation Mechanism of BLUF Photoreceptor Protein through Hybrid Quantum Mechanics/Molecular Mechanics Free-Energy Calculation. *ACS Phys. Chem. Au*, **2024**, *4*, 647-659.
